# MCA1 mechanosensitive channels enable fast communication of wound signals between lateral roots

**DOI:** 10.1126/sciadv.aef2202

**Published:** 2026-09-25

**Authors:** Angel Baudon, Craig R. Brodersen, Shouguang Huang, Huifang Song, Dirk Becker, Dietmar Geiger, M. Rob G. Roelfsema, Rainer Hedrich

**Affiliations:** ^1^ Molecular Plant Physiology and Biophysics, Julius-von-Sachs Institute for Biosciences, Biocenter, University of Würzburg, Julius-von-Sachs-Platz 2, 97082 Würzburg, Germany.; ^2^ School of the Environment, Yale University, New Haven, CT 06511, USA.; ^3^ Faculty of Synthetic Biology, Shenzhen University of Advanced Technology, Shenzhen, China.; ^4^ Institute of Emerging Agricultural Technology, Shenzhen University of Advanced Technology, Shenzhen, China.; ^5^ State Key Laboratory of Quantitative Synthetic Biology, Shenzhen Institute of Synthetic Biology, Shenzhen Institutes of Advanced Technology, Chinese Academy of Sciences, Shenzhen, China.

## Abstract

Although plant roots are hidden in soil, they are vulnerable to damage by insect herbivory, such as aerial tissues. However, wound signaling in roots is poorly understood. Here, we examined how damage signals spread locally and over long distances between *Arabidopsis* lateral roots. Using intracellular membrane potential recordings, calcium imaging, and optogenetics, we show that mechanical injury triggers an immediate local membrane depolarization and cytosolic calcium elevations whose magnitude and duration scale with wound severity. Depolarizations were also detected in neighboring lateral roots within milliseconds, demonstrating the presence of a rapid inter-root signaling pathway. Through mutant analyses, we highlight the roles of glutamate-like receptors and mid1-complementing activity 1 (MCA1) mechanosensitive channels in mediating this long-distance communication. Our results demonstrate that a wound-induced decrease in root cell turgor pressure rapidly spreads across the root network, where neighboring roots decode this signal via MCA1. This work underscores fundamental differences between root and shoot wound responses and uncovers a mechanosensory basis for fast communication between lateral roots.

## INTRODUCTION

Throughout evolution, plants have developed defense mechanisms to resist attacks by phytophagous animals, both in aboveground and belowground tissues (*1*). Although the root surface represents a crucial plant-to-environment interface and root damage impairs crop production, only a few studies have investigated root defense mechanisms. As a result, our knowledge of the plant root defense systems is sparse, and general concepts found in the shoot are often not transposable to roots (*2*). Therefore, filling this gap and understanding how plant roots detect and respond to damage caused by pathogens, such as nematodes and insects, is crucial for optimizing crop production.

In addition to bacteria, fungi, and nematodes that cause single-cell root wounding, insects and their larvae can mechanically damage large areas of roots. Neighboring root cells can detect the resulting cell debris as damage-associated molecular patterns (DAMPs) and activate defense mechanisms to protect themselves, such as the production of inducible physical and chemical barriers and toxic metabolites (*3*).

Besides the immediate local defense response at the wounded site, plants also show systemic responses that require communication within or between organs. Chemical or physical messengers can mediate this long-distance signaling. Many studies investigating plant stress signals in the shoot highlighted the importance of fast-propagating electrical and Ca^2+^ signals. For instance, caterpillar feeding on *Arabidopsis* leaves provokes long-distance leaf-to-leaf Ca^2+^ and electrical waves (*4*–*6*). In shoot tissues, several molecules have been identified as messengers for long-distance signaling, including glutamate (*6*) and the β-thioglucoside glucohydrolase (*7*). In addition, physical signals, such as variations in turgor pressure, appear to serve as signals transmitted among leaves, with mechanosensors functioning as their decoders (*8*).

In contrast to systemic signaling in shoots, the mechanisms underlying the initiation and propagation of Ca^2+^/electrical signals within the root system have received little attention so far. In the present study, we addressed this issue and showed that lateral root wounding provokes an immediate depolarization of epidermal cells, followed by a Ca^2+^ signal that spreads locally. At the same time, wounding also triggers depolarization of distant roots that involves glutamate receptor–like cation channels and mechanosensitive channels. Our results show that the drop in turgor pressure due to membrane disruption reaches distant sites within the root and opens mechanosensitive channels, generating a depolarization and a Ca^2+^ transient.

## RESULTS

### Lateral root wounding provokes epidermal cell depolarization and cytosolic Ca^2+^ signals

**Fig. 1.**
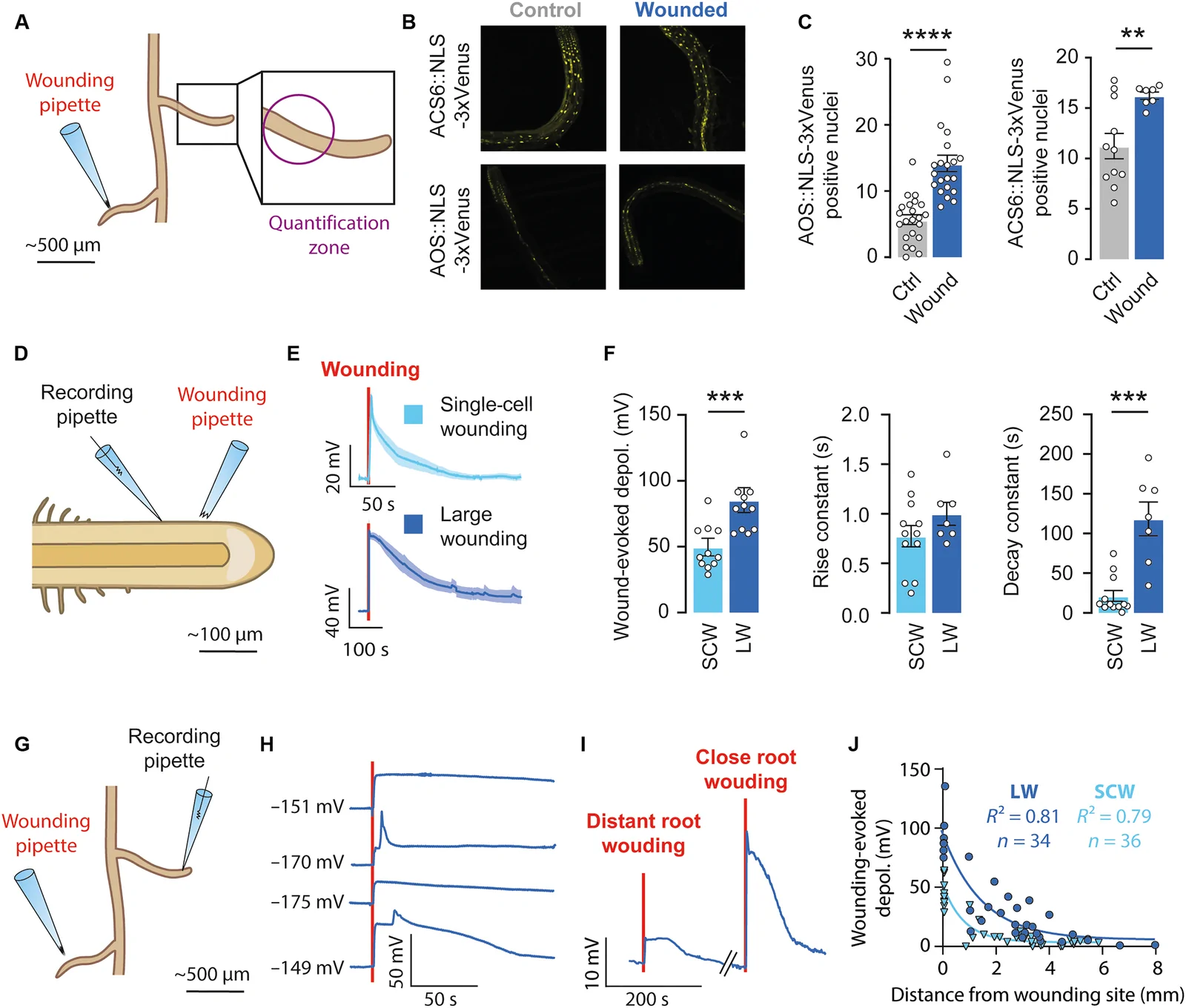
Wound-induced defense responses and electrical signals. (**A**) Lateral roots were wounded, and 5 hours later, ethylene and jasmonate responses were monitored in neighboring roots based on the fluorescence of Venus under the control of the *ACS6* or *AOS* promoter. The purple circle illustrates the 300-μm zone in which fluorescent nuclei were counted. (**B** and **C**) Example images and quantification of lateral roots showing nuclear Venus expression under the control of the *ACS6* (top) or *AOS* (bottom) promoter at control conditions (left; Ctrl) or after wounding a neighboring root (right; wound). *AOS*, *n*_Ctrl_ = 22 and *n*_Wound_ = 21; *ACS6*, *n*_Ctrl_ = 11 and *n*_Wound_ = 7. (**D**) Lateral root epidermal cells were impaled with sharp voltage-recording microelectrodes, and single-cell woundings were performed using sharp glass pipettes, or large wounds were made with ∼10-μm pipettes. (**E**) Mean membrane potential depolarization caused by single-cell wounding (SCW; light blue) or large wounding (LW; dark blue). The vertical red line marks the wounding time. (**F**) Characteristics of the depolarization evoked by single-cell wounding or large wounding. *n*_SCW_ = 12 and *n*_LW_ = 7. (**G**) Lateral root epidermal cells were impaled with voltage recording electrodes, and large wounds were inflicted on distant lateral roots. (**H**) Membrane potential depolarization caused by a distant large wound. Each trace is from a different plant; the initial membrane potential is given for each trace, and the red line indicates the wounding time. (**I**) Membrane potential depolarization triggered by wounding a distant and a nearby lateral root; the diagonal lines indicate an interruption of ∼600 s. (**J**) Depolarization amplitudes evoked by single-cell or large wounding plotted against the distance between the wounded root and the recording site. *n*_LW_ = 34 and *n*_SCW_ = 36. Data in (C) and (F) are mean across plants ± SEM. Detailed statistics are available in table S1. See also fig. S1. **P < 0.01, ***P < 0.001, and ****P < 0.0001.

The elongation zone (EZ) of lateral roots is a highly vulnerable tissue, as cells in this fast-growing part of the root system are not yet protected by a secondary cell wall (*2*). In response to wounding, this region activates the ethylene defense pathway and also the jasmonate pathway if the vascular tissue is damaged (*9*). Because vascular strands connect neighboring lateral roots, we examined whether ethylene and jasmonate pathways are also activated in roots adjacent to wounded lateral roots. We used 10- to 14-day-old seedlings that either express the ethylene reporter *ACS6::NLS-3xVenus* or the jasmonate reporter *AOS::NLS-3xVenus* (Fig. 1A). Wounding of a lateral root evoked a systemic ethylene and jasmonate response in neighboring lateral roots (Fig. 1, B and C), indicating that the wound signal is transmitted along the root system.

In the shoot, wounding triggers the propagation of electrical signals (*10*). What about the root? To answer this question, we performed single-cell micromanipulator-assisted mechanical wounding of the lateral root tip with <0.1-μm sharp glass microcapillaries, while the membrane potential was recorded with a glass electrode (Fig. 1D and fig. S1, A to C). Wounding a single cell in the root tip evoked an immediate depolarization of 49.4 ± 4.7 mV with a very fast rise (0.78 ± 0.11 s) and a decay of 21.5 ± 6.8 s (*n* = 12; Fig. 1, E and F). In contrast, damaging the entire cross section of the lateral root with glass capillaries that had a tip diameter of 10 μm resulted in an approximately two times larger depolarization (91.4 ± 6.3 mV, *P* < 0.0001) with a similar rise time (1.00 ± 0.12 s, *P* = 0.1921) but a much slower decay time (118.4 ± 21.2 s, *P* < 0.0001, *n* = 7; Fig. 1, E and F).

To test whether wound-evoked electrical signals propagate, we wounded a lateral root while measuring the EZ epidermal cell membrane potential of a neighboring lateral root (Fig. 1G). In all wounding experiments performed, we detected almost immediate (millisecond range) depolarizations at the distant site (Fig. 1H). The amplitudes of distant wounding-evoked depolarizations were variable and appeared to decrease with the distance between the recording and the wounding site. To resolve the amplitude-distance relation, we impaled a lateral root and performed the consecutive wounding of two different neighboring lateral roots, one more distant from the recorded root, and another one closer to the recording site. The depolarization observed following a distant root wounding was considerably smaller compared to the one caused by wounding of a more adjacent lateral root (Fig. 1I), suggesting that the depolarization amplitude depends on the distance. On average, the wound-evoked depolarization amplitude decayed exponentially with the distance between the wounding and the recording site (Fig. 1J). Single-cell wounding-evoked depolarization was detected up to 2 mm away from the wounding site and had a half-maximum distance of 0.55 mm (Fig. 1J). When a large wound was imposed, we could detect the evoked depolarization up to 5 mm away from the wounding site (half-maximum distance = 1 mm; Fig. 1J).

### Wounding evokes Ca^2+^ signaling in roots

**Fig. 2.**
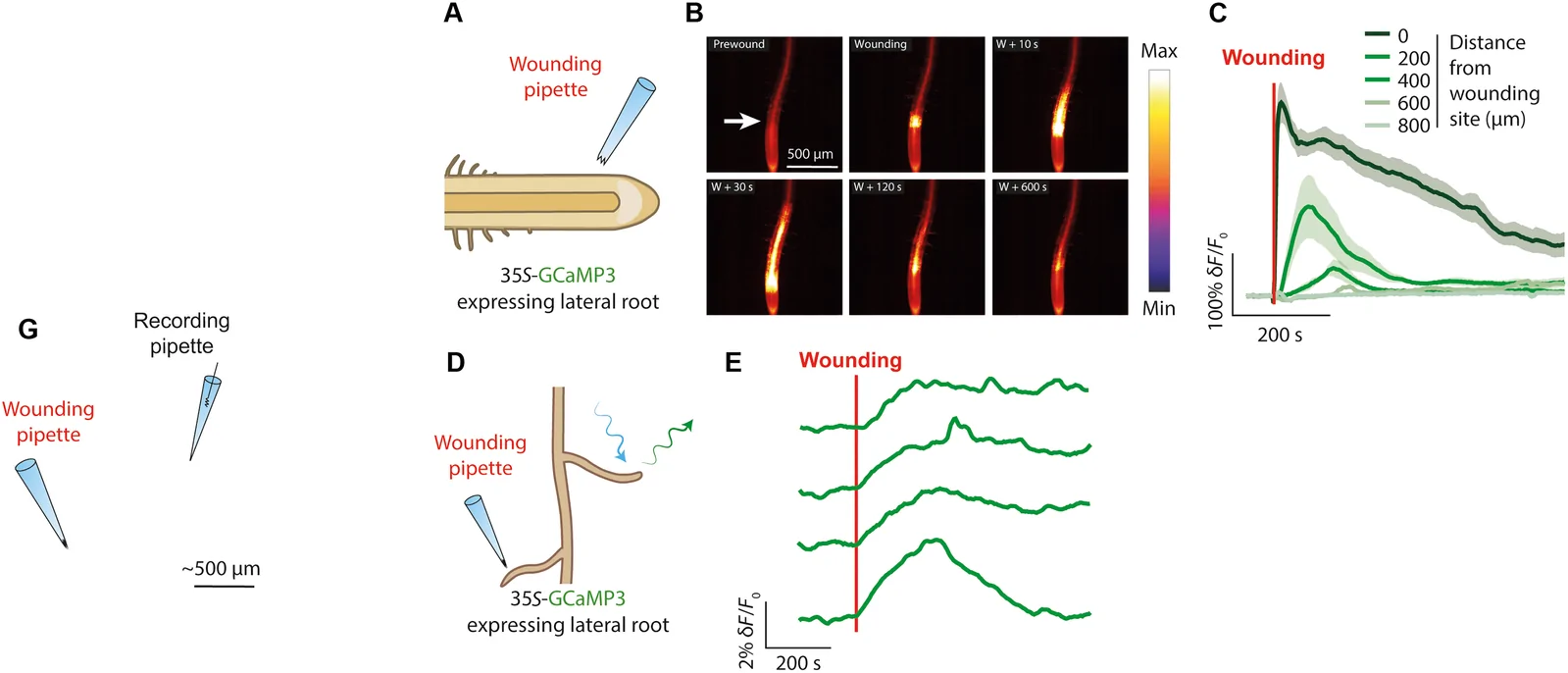
Lateral root wounding-evoked Ca^2+^ signals. (**A**) The lateral root fluorescence of a GCaMP3-expressing seedling was recorded, and a local wound was performed with a large ∼10-μm glass pipette. (**B**) Pseudo-colored images showing lateral root GCaMP3 fluorescence intensity at time points before, during, and after wounding. The white arrow indicates the wound site. *n* = 8. (**C**) Average fluorescence levels of GCaMP3 measured at several distances from the wound site. The red line indicates the time point of the wounding. (**D**) The GCaMP3 fluorescence intensity was monitored in one lateral root, while a neighboring lateral root was wounded. (**E**) Wound-induced changes of GCaMP3 fluorescence intensity plotted against time. Each trace is from an individual experiment. The red line indicates the time point of wounding. Data are expressed as mean values ± SEM. Detailed statistics can be found in the table S2. See also fig. S2.

Electrical signals and cytosolic Ca^2+^ elevations are both evoked during leaf wounding (*4*, *8*). We therefore monitored cytosolic Ca^2+^ signals in lateral roots that express the genetically encoded Ca^2+^ indicator GCaMP3. Fluorescence imaging of GCaMP3-expressing seedlings showed that lateral root single-cell wounding triggered a 391 ± 27% fluorescence increase that reached a peak within 12 ± 2 s and decayed in 182 ± 50 s (fig. S2, A and B). Increasing the wounding size resulted in a Ca^2+^ transient with a similar amplitude (361 ± 28%, *P* = 0.4521) and a similar rise time (22 ± 7 s, *P* = 0.2606) but a slower decay (391 ± 27 s, *P* = 0.0023; Fig. 2, A to C, and fig. S2, A to C). This response suggests that the duration, rather than the amplitude, of the Ca^2+^ transient reports the strength of the injury.

When measuring the spread of wounding-evoked Ca^2+^ signal in lateral roots, we observed that single-cell wounding-evoked Ca^2+^ transients did not propagate very far (half-maximum distance = 91 μm; fig. S2, A to C). Ca^2+^ signals generated by large wounding propagated further but remained relatively close to the wounded area (half-maximum distance = 193 μm; Fig. 2, A to C). These observations are in stark contrast to what has been observed in the shoot, where a strong and nondecremental Ca^2+^ wave propagates from the wounded leaf to interconnected leaves (*6*). High-resolution Ca^2+^ measurements with GCaMP3 in neighboring roots (Fig. 2D) revealed that wounding also caused Ca^2+^ concentration changes in the EZs of roots adjacent to the wounded site (Fig. 2E).

### DAMPs elicit local and long-distance electrical signaling

**Fig. 3.**
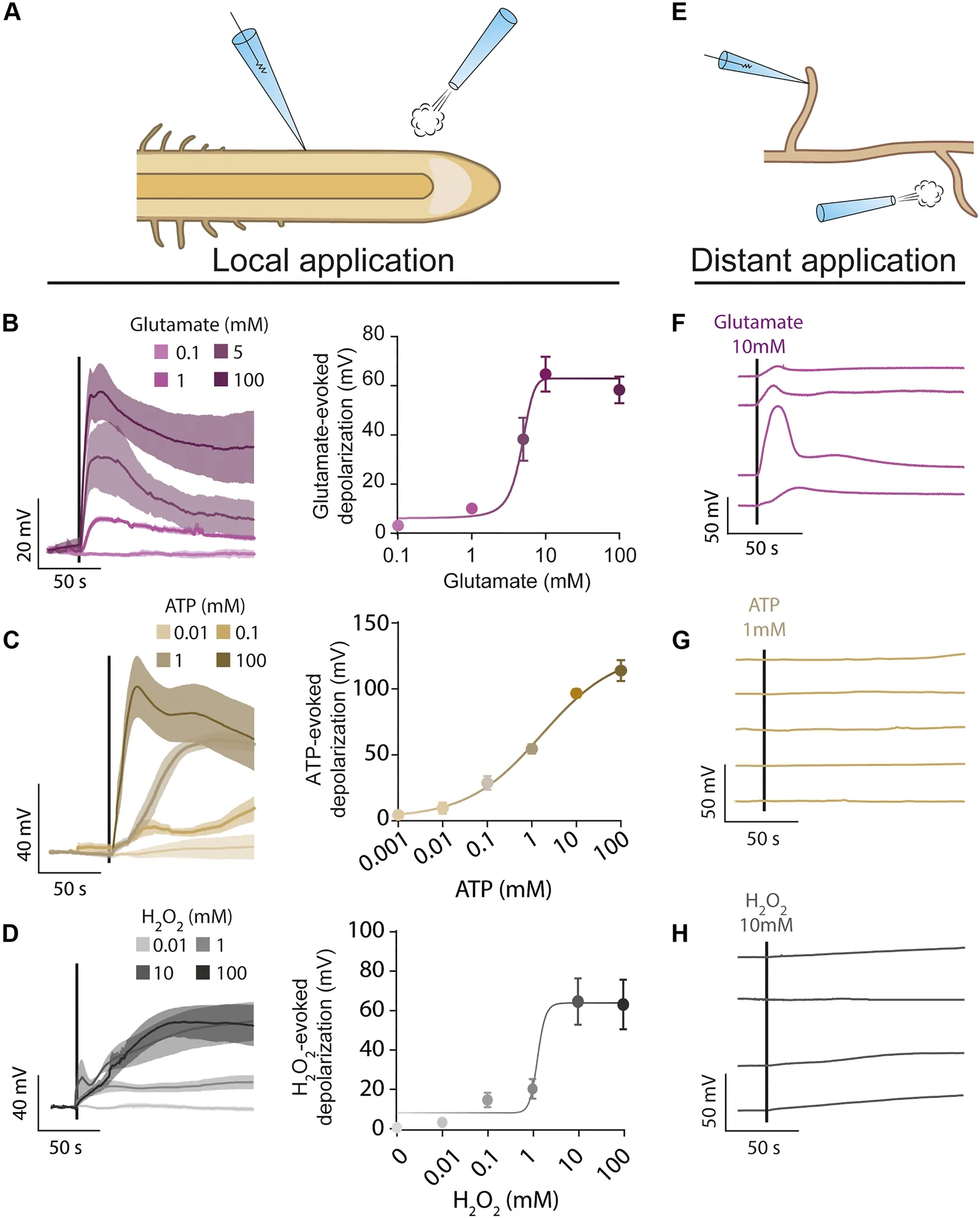
Effect of DAMPs on root membrane potential. (**A**) Epidermal cells of lateral roots were impaled with voltage recording electrodes, and a solution containing glutamate (**B**), ATP (**C**), or H_2_O_2_ (**D**) was pressure applied locally with a second glass pipette over a period of 10s. [(B) to (D)] Average membrane potential traces of root cells stimulated with different concentrations of DAMPs (left) and corresponding quantification (right). (**E**) Epidermal root cells of lateral roots were impaled with a voltage recording electrode, and a solution containing glutamate (**F**), ATP (**G**), or H_2_O_2_ (**H**) was pressure applied on a neighboring root with a second glass pipette for 10 s. [(F) to (H)] Membrane potential traces of individual epidermal root cells during a distant DAMP application. Black lines indicate the time point of DAMP application. Data in (B) to (D) are expressed as mean values ± SEM. Detailed statistics can be found in the table S3.

Following wounding, DAMPs can be released from wounded cells and convey the wounding information (*11*). To test for DAMP responses, we locally applied glutamate, adenosine triphosphate (ATP), and H_2_O_2_. DAMPs stimulation was performed with glass application pipettes connected to a pressure ejection system, while we used voltage-recording intracellular electrodes to monitor the responses of EZ cells in lateral roots (Fig. 3A). Locally, all three DAMPs elicited robust depolarizations (Fig. 3, B to D) with similar median effective concentration values (glutamate, 4.7 mM; ATP, 1.6 mM; H_2_O_2_, 3.1 mM). However, the rise kinetics of the depolarization were different, with glutamate triggering a faster membrane potential increase of epidermal root cells, compared to the other DAMPs (glutamate, 22.1 ± 4.8 s; ATP, 104.9 ± 24.9 s; H_2_O_2_, 99.67 ± 19.74 s; *P* = 0.0048; Fig. 3, B to D).

To test whether the DAMP-mediated depolarizations can travel from one lateral root to another, we recorded the membrane potentials of root cells while applying DAMPs on a neighboring lateral root (Fig. 3E). Under these conditions, neither H_2_O_2_ nor ATP was able to generate membrane potential depolarizations in the distant lateral root. However, glutamate did trigger depolarizations in distant lateral roots (Fig. 3, F to H), pointing to a key role of glutamate in wound-evoked communication between lateral roots.

### Glutamate-like receptors participate in long-distance wound signaling in roots

**Fig. 4.**
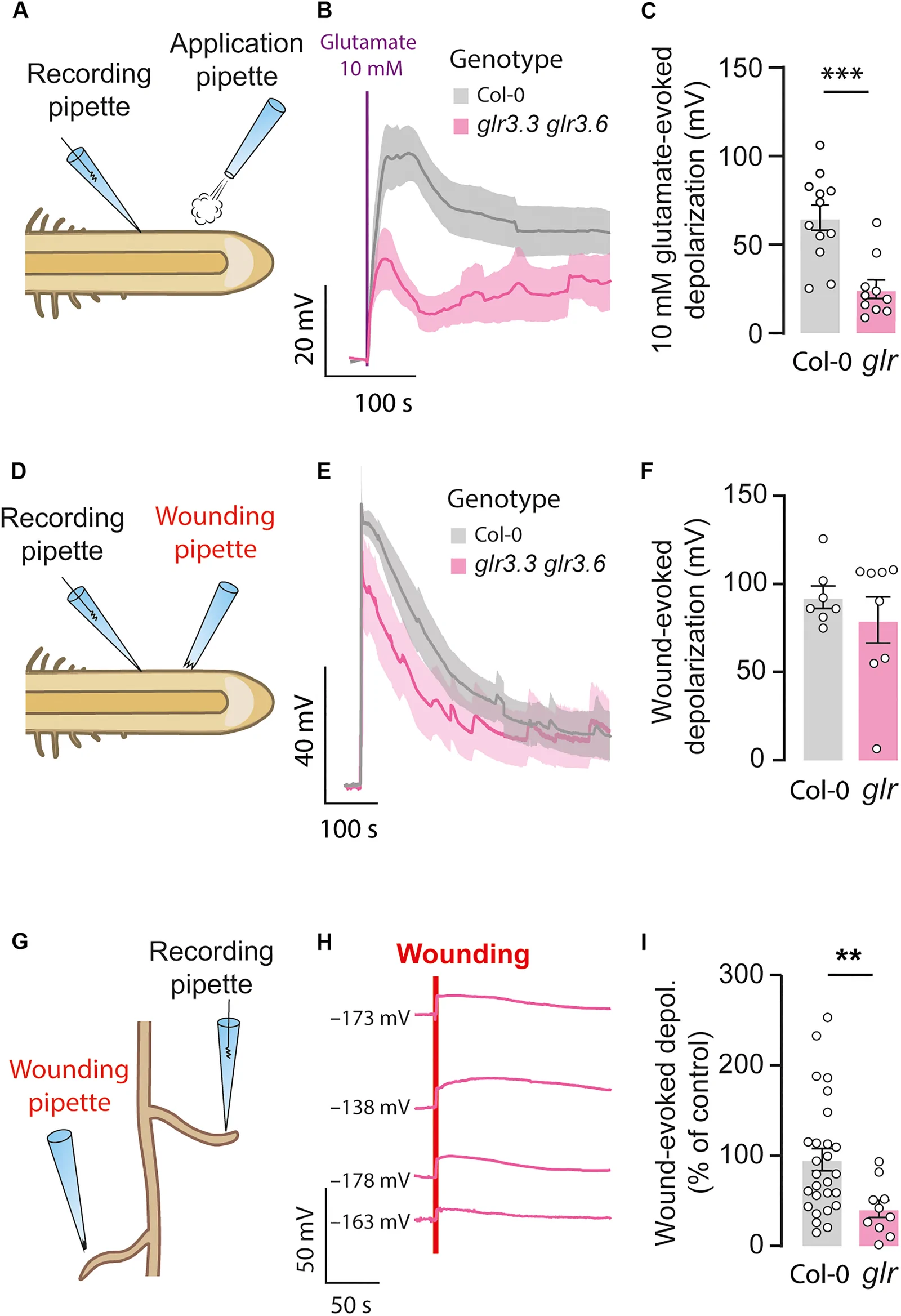
GLRs are important for long-distance wound-evoked depolarizations. (**A**) Epidermal root cells were impaled with a voltage recording electrode and a solution containing 10 mM glutamate was pressure applied with a second glass pipette for 10 s. (**B**) Mean traces of the glutamate-evoked depolarization. The time point of glutamate application is indicated with the purple line; the gray traces correspond to wild type, and the pink ones to the *glr3.3/3.6* mutant. (**C**) Amplitude of glutamate-evoked depolarization. *n*_Col-0_ = 12 and *n*_glr_ = 10 plants. (**D**) Lateral root epidermal cells were impaled with voltage recording electrodes, and large wounds were inflicted. (**E**) Mean depolarization of the membrane potential evoked by a large wound in wild type (gray) or *glr3.3/3.6* mutant (pink). The magenta line indicates the time point of wounding. (**F**) Quantification of the wound-evoked depolarization amplitude, rise, and decay constants. *n*_Col-0_ = 7 and *n*_glr_ = 8 plants. (**G**) The epidermal cell of a lateral root was impaled with a voltage recording electrode, and a large wound was performed on another, distant lateral root. (**H**) Traces of membrane potential depolarization of the *glr3.3/3.6* mutant, evoked by a large wounding of a distant lateral root. Each trace comes from a different plant; the number before the trace indicates the resting membrane potential, and the red line indicates the time point of wounding. (**I**) Amplitude of distant wound-evoked depolarization recorded in control and *glr3.3/3.6* mutant plants, normalized by their predicted amplitude based on the distance between the recorded site and the wounding point. *n*_Col-0_ = 27 and *n_glr_* = 10. Data in (B), (C), (E), (F), and (I) are expressed as mean across plants ± SEM. Detailed statistics can be found in the table S4. See also fig. S4. **P < 0.01 and ***P < 0.001.

Glutamate-like receptors (GLRs), and especially GLR3.3 and GLR3.6, represent potential candidates to detect glutamate, since they are involved in leaf-to-leaf propagation of wounding-evoked Ca^2+^ and electrical signals (*4*, *10*). To probe the function of these GLRs in the root, we impaled root epidermal cells of seedlings lacking functional GLR3.3 and GLR3.6 (*glr3.3/3.6*). Local depolarization evoked by glutamate application was reduced in roots of *glr3.3/3.6* mutant plants (Fig. 4, A to C, *P* > 0.001). To test the involvement of these GLRs in long-distance propagation of glutamate-evoked depolarization, we impaled epidermal cells of a lateral root and applied glutamate to a distant lateral root. In plants lacking functional GLR3.3/3.6, application of glutamate on a distant root only triggered depolarizations of low amplitude (fig. S4D). Together, these data make GLRs potential candidates for conveying wounding-associated DAMP-mediated depolarization in the root.

Given that wounding is a complex event that likely involves different DAMPs, we wondered whether impairing components of the glutamatergic system in the root was enough to prevent wound signaling. In *glr3.3/3.6* mutant plants, the amplitude and kinetics of local wound-evoked depolarization were similar to those observed in Col-0 control plants (*P*_amplitude_ > 0.999, Fig. 4, D to F; *P*_rise_ = 0.795 and *P*_decay_ = 0.235, fig. S4E). This behavior suggests that GLR3.3 and GLR3.6 are not key in the generation of the local depolarization evoked by root wounding, as shown before (*12*). What about the distal sites? To answer this question, we tested whether the selected GLRs play a major role in wounding-evoked depolarization at sites distal to the wounding site (Fig. 4G). Following wounding, we observed small depolarizations of the membrane potential in *glr3.3/3.6* mutant plants (Fig. 4H). To evaluate whether these depolarizations were smaller than in wild-type plants, we inferred their expected amplitude from their distance using the exponential relationship in Fig. 1J. This analysis indicated that wounding-evoked depolarizations observed in *glr3.3/3.6* mutants were significantly smaller (41 ± 9%; Fig. 4I) compared to those expected for the same distance in wild type (*P* = 0.0058). Together, these findings indicate that GLRs are involved in the propagation of electric signals from the site of wounding to neighboring lateral roots.

### Wound-evoked hydrostatic pressure drop activates mechanosensitive channels

**Fig. 5.**
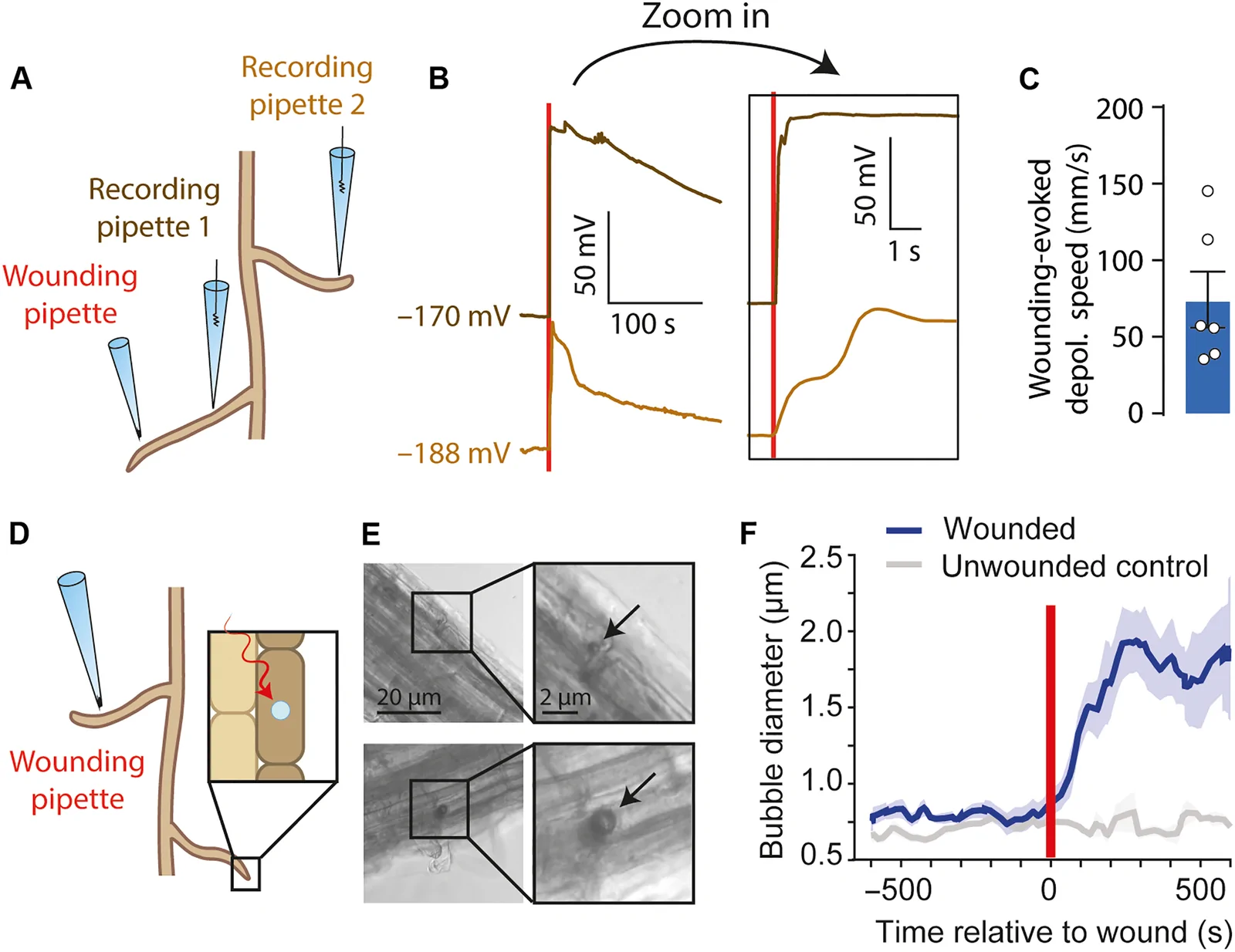
Wound-evoked pressure drop travels between lateral roots at high speed. (**A**) Epidermal cells from two neighboring lateral roots were impaled with voltage-recording microelectrodes, and one of them was wounded. (**B**) Simultaneous recording of membrane potentials in the wounded root (dark brown; recording pipette 1) and in another lateral root (light brown; recording pipette 2). The inset shows the same membrane potential traces at a shorter timescale. The red line indicates the wounding time. (**C**) Propagation speed of the wound-evoked depolarization. *n* = 6. (**D**) Intracellular pressure monitoring using cavitation bubbles generated in epidermal cells with a laser before and after wounding a neighboring lateral root. (**E**) Micrographs of cavitation bubbles. (**F**) Changes in diameter of the cavitation bubbles plotted against time. A neighboring lateral root was wounded at the time point indicated by the red line. *n*_Ctrl_ = 6 and *n*_Wound_ = 7. Detailed statistics can be found in the table S5.

Taking a closer look at distant wound-evoked depolarizations, we observed an extremely short delay between the wounding and the depolarization recorded at the distant recording sites (Fig. 1, H and I). To measure precisely the speed of the wounding-evoked electrical signal, we simultaneously recorded the membrane potential of cells in two lateral roots and wounded one of them (Fig. 5A). On the basis of the delay between the onsets of the wounding-provoked electrical signals, we calculated a mean speed of 74.7 ± 18.2 mm/s (Fig. 5, B and C). In comparison, the speed of the wound-evoked Ca^2+^ wave observed near the wounding site (Fig. 5, B and C) was 2.59 × 10^−3^ mm/s and thus about four orders of magnitude slower than the depolarization response. This observation indicates that a signal travels several centimeters per second and generates a wounding-associated depolarization in distant lateral roots.

Which biologically relevant signal can travel that fast? Plant cells represent a hydrodynamic system that is poised to sense changes in hydrostatic pressure. Given that pressure waves can travel at high speed, we hypothesize that the sudden loss of turgor pressure due to wounding could propagate to interconnected neighboring cells and pipes such as the xylem/phloem network. To measure the internal pressure in epidermal root cells without damaging them, we used the recently developed cavitation bubble manometry (*13*), where a laser is used to nucleate a microscopic cavitation bubble inside plant cells. By monitoring the formation and maximum diameter of the resulting microbubbles with a high-speed camera, we could monitor changes in the lateral root cell’s turgor pressure (Fig. 5D). This showed that the pressure within undamaged lateral roots remained relatively stable over 20 min, while wounding a neighboring lateral root triggered a diameter increase in the laser-generated microbubble, indicating a drop of the turgor pressure (Fig. 5, E and F). This direct measure of intracellular pressure thus strongly suggests that wound-mediated pressure drops can travel from one lateral root to its neighbors.

How can this pressure drop serve as a signal, and how is it decoded by root cells? To mimic this drop of turgor pressure without rupturing the plasma membrane, we locally applied a hyperosmotic solution of sorbitol for a very short time (1 s). When applying 500 mM sorbitol, the recorded EZ epidermal cells immediately depolarized by 111.5 ± 4.6 mV (fig. S5, A and B). These sorbitol-mediated hyperosmotic shocks were also sufficient to trigger local Ca^2+^ signals (fig. S5, C and D), which indicates that mechanosensitive elements sense changes in the root cells’ pressure and generate a Ca^2+^ signal.

**Fig. 6.**
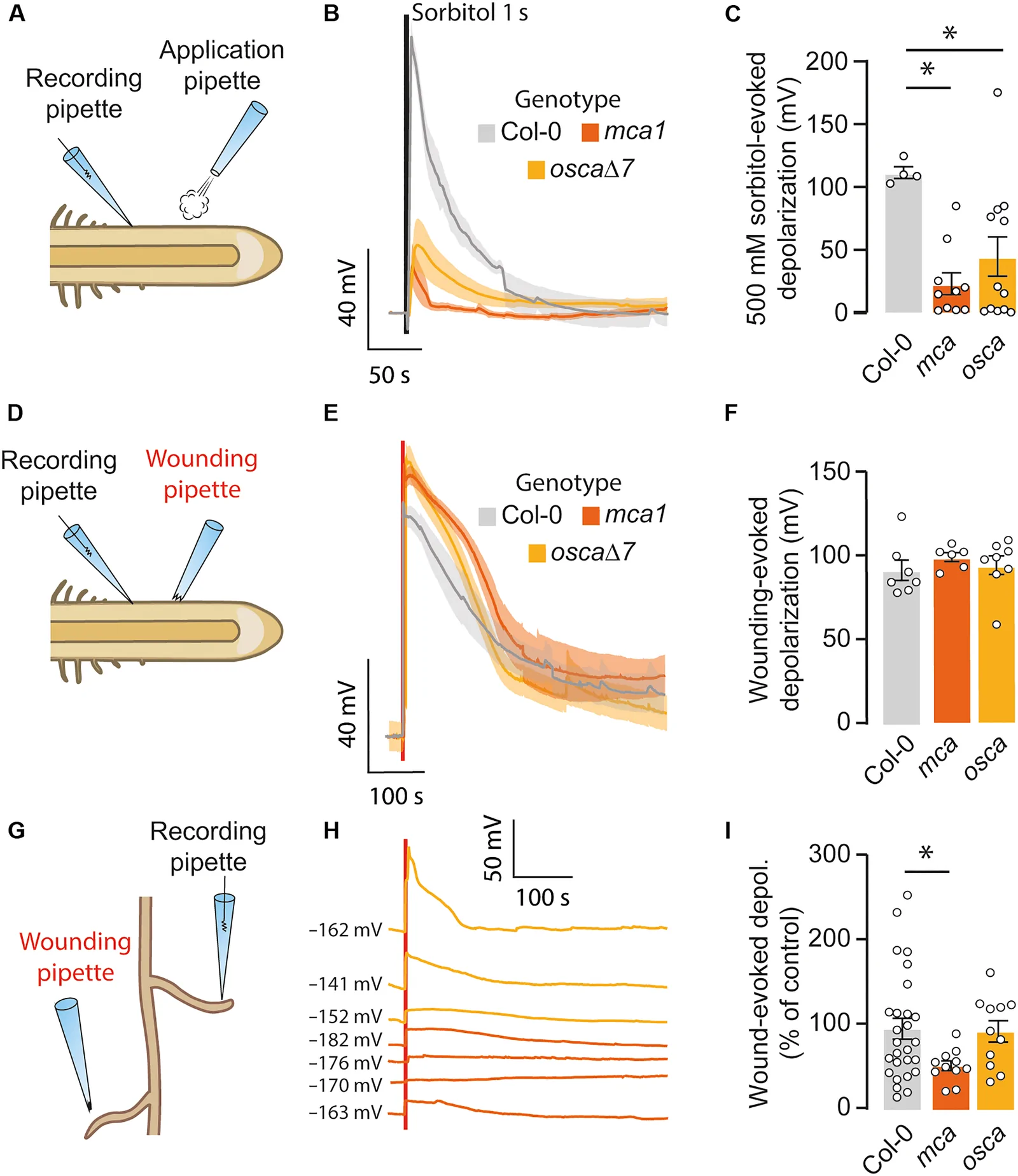
Involvement of mechanosensitive cation channels in wound signaling. (**A**) Epidermal root cells were impaled, and a solution containing sorbitol was locally applied with a second glass pipette for 1 s. (**B** and **C**) Mean traces and amplitude of sorbitol-evoked depolarization. The black line indicates the sorbitol application time. *n*_Col-0_ = 4, *n*_mca1_ = 10, and *n*_osca1_ = 12. (**D**) Epidermal cells were impaled, and large wounds were made locally. (**E** and **F**) Mean trace and amplitude of local wound-evoked depolarization in wild-type, *mca1*, or *osca1* mutants. The red line indicates the wounding time. *n*_Col-0_ = 7, *n*_mca1_ = 6, and *n*_osca1_ = 8. (**G**) The epidermal cell of a lateral root was impaled, and a large wound was provoked on another distant lateral root. (**H**) Mean trace of distant wound-evoked depolarization in wild-type, *mca1*, or *osca1* mutants. Each trace comes from a different plant; the number before the trace indicates the resting membrane potential, and the red line indicates the wounding time. (**I**) Amplitude of distant wound-evoked depolarization recorded in control, *mca1*, or *osca1* mutant plants normalized by their predicted amplitude based on the distance between the recorded site and the wounding point. *n*_Col-0_ = 27, *n*_mca1_ = 11, and *n*_osca1_ = 11. Data are expressed as mean across plants ± SEM. Detailed statistics can be found in the table S5. See also fig. S5. *P < 0.05.

In plants, several types of mechanosensitive plasma membrane ion channels have been identified, such as MSL-type anion channels, OSCA-type, and MCA-type Ca^2+^-conductive cation channels (*14*). Since Ca^2+^ ions are probably involved in wound-mediated depolarization (Fig. 2), we investigated the role of the Ca^2+^-permeable mechanosensitive channels mid1-complementing activity 1 (MCA1) and OSCA1 and recorded membrane potential changes in lateral roots of loss-of-function mutants in response to local application of a 500 mM sorbitol solution (Fig. 6A). While application of sorbitol elicited a strong depolarization in wild-type plants (111.5 ± 4.6 mV, *n* = 4), the same stimulus provoked a much weaker depolarization in *osca1* and *mca1* mutant plants (*mca1*: 23.1 ± 8.8 mV, *n* = 10, *P* = 0.011; *osca1*: 44.7 ± 15.7, *n* = 12, *P* = 0.022; Fig. 6, B and C). Thus, OSCA1 and MCA1 can translate the turgor pressure drop into a membrane depolarization.

During a wounding event, the plasma membrane breaks, and the turgor pressure of the cell is lost. To test whether OSCA1 and MCA1 channels are involved in the wounding-induced local depolarization, we monitored the membrane potential of lateral roots (Fig. 6D). However, no difference in the amplitude and kinetics of the wound-depolarization in *osca1* and *mca1* plants compared to wild type was observed (Col-0: 92 ± 6.0 mV, *n* = 7; *mca1*: 98.8 ± 2.7 mV, *n* = 6, *P* = 0.265; *osca1*: 94.0 ± 15.7, *n* = 8, *P* = 0.523; Fig. 6, E and F). Thus, although MCA1 and OSCA1 are involved in turgor sensing, they do not mediate the depolarization following local wounding.

We hypothesized that a wound-mediated pressure drop sets off a signal that travels from a wounded lateral root to a distal one. This proposed pressure wave could activate mechanosensitive channels that depolarize cells at the distal site (Fig. 6G). After wounding a distant lateral root, we observed a reduced depolarization amplitude in *mca1*, but not in *osca1* mutant (*mca1*: 38.2 ± 6.8%, *P* = 0.0075; *osca1*: 92.1 ± 12.4%, *P* > 0.999; Fig. 6, H and I). This indicates that MCA1, but not OSCA1, channels are involved in decoding the mechanical long-distance signals from the wounding site to neighboring lateral roots.

### Long-distance wound-evoked Ca^2+^ signal relies on MCA1 and GLRs

**Fig. 7.**
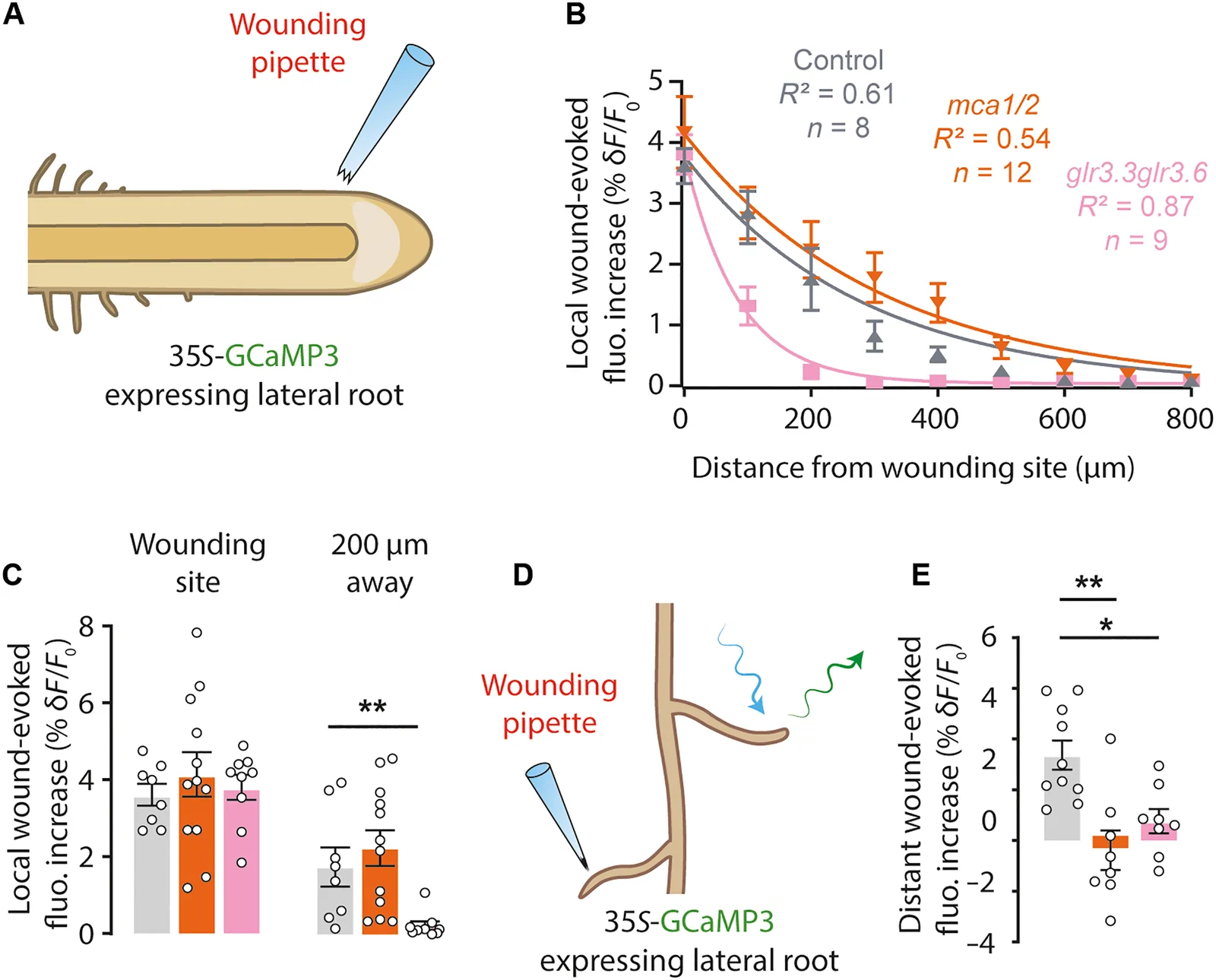
Involvement of MCA1/2, GLR3.3, and GR3.6 in wound-evoked Ca^2+^ signaling. (**A**) Lateral roots from GCaMP3-expressing seedlings were wounded with large ∼10-μm glass pipettes. (**B**) Plot showing the amplitude of the Ca^2+^ transient at different distances from the wounding site in control seedlings (gray), *mca1/2* (orange), or *glr3.3/3.6* mutants (pink). (**C**) Amplitude of Ca^2+^ transients recorded at the wounding site or at 200 μm. *n*_Ctrl_ = 8, *n*_mca_ = 12, and *n*_glr_ = 5 plants. (**D**) Lateral roots of GCaMP3-expressing seedlings were wounded, and GCaMP3 fluorescence was recorded in a neighboring lateral root. (**E**) Amplitude of the Ca^2+^ increase observed following the wounding of a neighboring lateral root recorded in control seedlings (Ctrl; gray; *n* = 10), in *mca1/2* mutants (orange; *n* = 8), or in *glr3.3/3.6* mutants (pink; *n* = 8). Data are expressed as mean values ± SEM. Detailed statistics can be found in the table S6. *P < 0.05 and **P < 0.01.

Given the Ca^2+^ channel nature of MCA1, we suggested that it should decode the pressure wave by translating it into a small, membrane-localized Ca^2+^ transient, which could then be amplified by GLRs. To test this hypothesis, we recorded wound-evoked Ca^2+^ transients in control plants and in loss-of-function mutants for MCA1/2 or for GLR3.3/3.6, all expressing GCaMP3 (Fig. 7A). Within the wounded root, the amplitude of the Ca^2+^ transient at the wounding site was not different between the three genotypes, indicating a similar GCaMP3 expression in these plants. The spatial spread of this high-amplitude Ca^2+^ wave, however, showed a noticeable difference: Although control and *mca1/2* showed a half-maximum distance of 193 and 214 μm, respectively, the Ca^2+^ wave was restricted in *glr3.3/3.6* plants, as quantified by its half-maximum distance of 66 μm (Fig. 7, B and C). Therefore, we propose that GLRs are involved in the local cell-to-cell propagation of the Ca^2+^ transient, possibly via Ca^2+^-induced Ca^2+^ release (*15*).

To investigate the involvement of these channels in the long distance–traveling small Ca^2+^ wave, we used these plants to record GCaMP3 fluorescence at high magnification of a lateral root while wounding a neighboring one (Fig. 7D). The wound-evoked Ca^2+^ transient was drastically reduced in *mca1/2* or *glr3.3/3.6* mutants compared to controls (Fig. 7E). This result suggests that MCA1 could translate the pressure wave into a small initial Ca^2+^ transient that is then amplified by GLR3.3 and GLR3.6.

### Priming Ca^2+^ signals represses wounding-evoked electrical responses

**Fig. 8.**
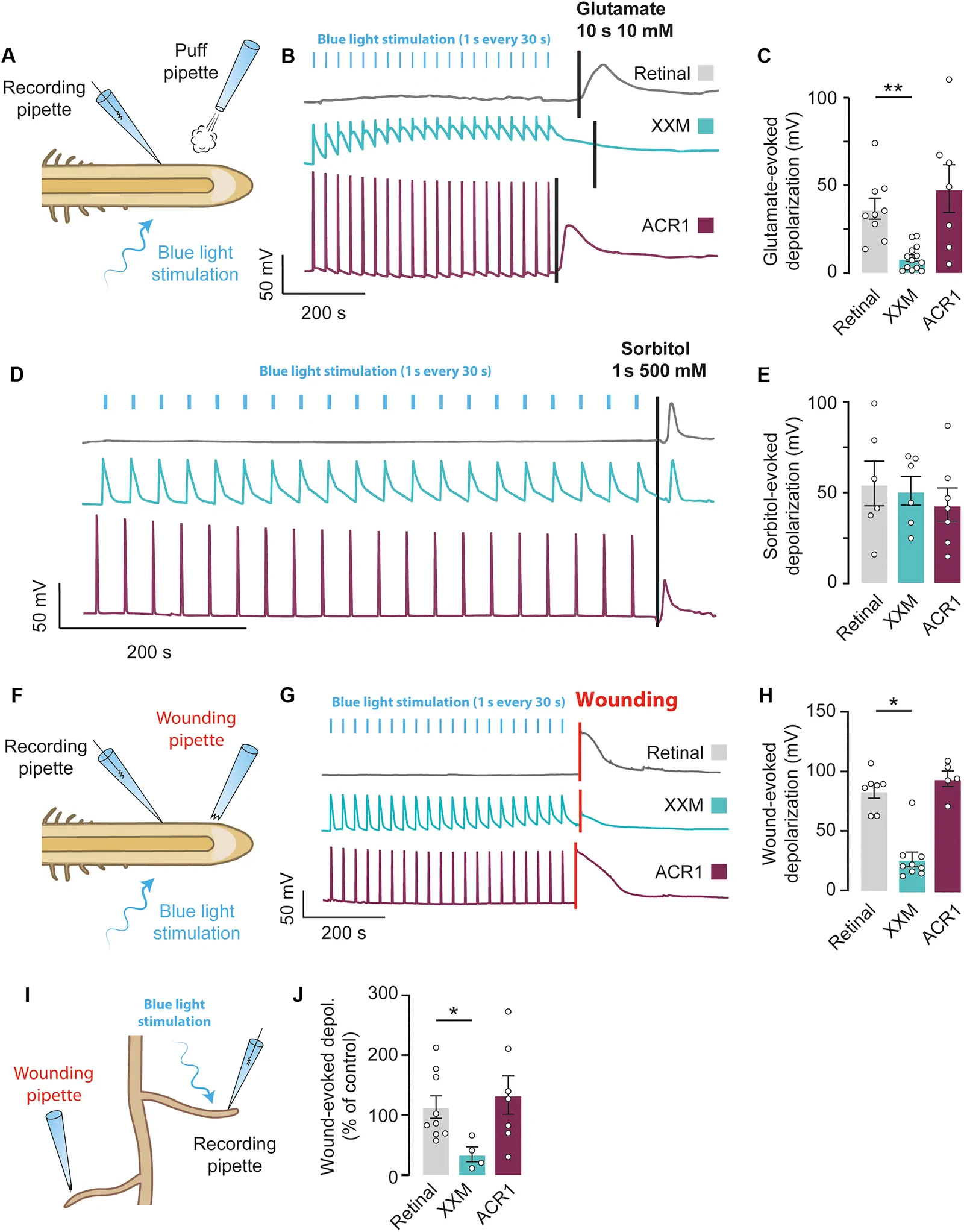
Optogenetic Ca^2+^ priming suppresses wound-evoked lateral root depolarization. (**A**) Lateral root epidermal cells were impaled with voltage recording electrodes and stimulated by a blue light train stimulation (20 pulses of 1 s spaced by 30 s). Sorbitol (500 mM, 1 s) or glutamate (10 mM, 10s) was locally applied with a second glass pipette. (**B** and **D**) Membrane potential traces recorded in seedlings expressing the retinal-synthesizing enzyme *Mb*DIO (gray), with the cation-permeable channelrhodopsin ChR2 XXM2.0 (turquoise), or the anion-permeable *Gt*ACR1 (plum). The light blue lines indicate the time points of light stimulations, and the black line indicates the application time point of sorbitol (B) or glutamate (D). (**C** and **E**) Amplitude of sorbitol-evoked (C) or glutamate-evoked (E) depolarization following the repetitive blue light pulses. Sorbitol: *n*_retinal_ = 9, *n*_XXM2.0_ = 11, and *n*_ACR1_ = 8; glutamate: *n*_retinal_ = 6, *n*_XXM2.0_ = 8, *n*_ACR1_ = 6. (**F**) Lateral root epidermal cells were impaled with voltage-recording electrodes and stimulated by a train of blue light pulses (20 pulses of 1 s spaced by 30 s). The same root was wounded ∼30 s following the train of light pulses. (**G**) Membrane potential traces recorded in seedlings expressing *Mb*DIO (gray), ChR2 XXM2.0 (turquoise), or *Gt*ACR1 (plum). The light blue lines indicate the time points of light stimulations, and the red line indicates the time of wounding. (**H**) Amplitude of wounding-evoked depolarizations in seedlings expressing retinal (*n* = 7) in combination with ChR2 XXM2.0 (*n* = 9) or *Gt*ACR1 (*n* = 5). (**I**) Epidermal cells of lateral roots were impaled and stimulated with blue light pulses before wounding neighboring lateral roots. (**J**) Amplitude of distant wound-evoked depolarization normalized by its predicted amplitude based on the distance between the recorded site and the wounding point. Retinal (*n* = 9), ChR2 XXM2.0 (*n* = 6), or *Gt*ACR1 (*n* = 7). Data in (C), (E), (H), and (J) are expressed as mean across plants ± SEM. Detailed statistics can be found in the table S7. *P < 0.05 and **P < 0.01.

Recent work has shown that GLR3.3 is inhibited by a Ca^2+^/calmodulin module (*16*). Given the involvement of this GLR in wound signaling (Figs. 4 and 7), we wondered whether priming the root with Ca^2+^ transients feeds back on root response to glutamate and wounding. To test this assumption, we generated *Arabidopsis* plants expressing the light-activated Ca^2+^-permeable ChR2 XXM2.0 channel to impose Ca^2+^ transients noninvasively (*15*, *17*–*19*), while measuring the membrane potential of EZ root cells with intracellular voltage-recording microelectrodes (Fig. 8A).

Seedlings expressing ChR2 XXM2.0 and retinal cofactor–generating dioxygenase (*20*) under the *UBQ10* promoter were primed with a train of blue light pulses (1 s every 30 s, 20 pulses). This light stimulation triggered robust and reproducible depolarizations, which were absent in seedlings expressing only the retinal cofactor without ChR2 XXM2.0 (Fig. 8B). Following this priming stimulus, we locally applied glutamate with a puff pipette and monitored the resulting depolarization. Although glutamate application evoked robust membrane potential depolarization in control plants expressing only the retinal cofactor, this effect was almost absent in XXM2.0-expressing lateral roots (retinal, 37.6 ± 6.6; XXM2.0, 6.9 ± 1.7; *P* = 0.0042; Fig. 8, B and C). This result indicates that Ca^2+^ priming impairs local glutamate-mediated depolarization. However, ChR2 XX2.0 also triggers large depolarization, which can be a confounding factor. To test this possibility, we used the anion channelrhodopsin *Gt*ACR1 as a depolarization control (*19*, *20*). In seedlings expressing *Gt*ACR1, a train of blue light pulses (1 s every 30 s, 20 pulses; Fig. 8B) repeatedly depolarized the membrane potential of epidermal root cells (Fig. 8, B and C). However, this train of depolarizations did not affect the depolarization induced by glutamate application (retinal, 37.6 ± 6.6 mV; ACR1, 49.6 ± 10.8 mV; *P* = 0.3737; Fig. 8, B and C). Therefore, repetitive Ca^2+^ transients, but not repetitive depolarizations, reduce the amplitude of glutamate-evoked root depolarization.

Since MCA1 is also involved in wound signaling, we also checked whether Ca^2+^ priming desensitizes this channel. Application of sorbitol to plants previously primed with ChR2 XXM2.0 or *Gt*ACR1 optogenetic activation elicited a depolarization response similar to the one observed in control plants (*P* = 0.6539; Fig. 8, D and E). The unchanged perception of pressure drop indicates that the repetitive Ca^2+^ influx triggered by ChR2 XXM2.0 does not desensitize MCA1.

Having shown that Ca^2+^ priming reduces glutamate perception in lateral roots, we wondered whether it could also influence wounding perception. Following a train of blue light pulses, local wounding of the root triggered a smaller membrane potential depolarization in ChR2 XXM2.0–expressing plants compared to the one observed in retinal-expressing controls (XXM2.0, 27.3 ± 6.2; retinal, 83.6 ± 6.0; *P* = 0.0192; Fig. 8, F to H). Repetitive light stimulation of the GtACR1-expressing depolarization control did not affect the depolarization caused by wounding (ACR1, 94.11 ± 6.46 mV; retinal, 83.6 ± 6.0 mV; *P* > 0.999; Fig. 8, F to H). Given that repetitive Ca^2+^ transients, but not repetitive depolarizations, reduce the amplitude of the local wounding-evoked root depolarization, this indicates that Ca^2+^ signals prime the root to be less sensitive to subsequent wounding.

To test whether Ca^2+^ manipulation also disrupts the depolarization observed in lateral roots adjacent to the wounded one, we recorded the membrane potential of lateral roots and photostimulated them before wounding another lateral root (Fig. 8I). Wounding a distant lateral root after Ca^2+^ priming via ChR2 XXM2.0 triggered a smaller depolarization compared to controls (63.4 ± 19.9% of the control; Fig. 8J). In contrast, depolarization priming of the local root via light stimulations of *Gt*ACR1-expressing seedlings did not affect the amplitude of depolarization triggered by distant root wounding (133.3 ± 31.9% of the control; Fig. 8J). This finding indicates that repetitive Ca^2+^ transients decrease the wounding-associated depolarization, both in the wounded root and in neighboring lateral roots, probably by desensitizing GLR3.3 activity.

## DISCUSSION

**Fig. 9.**
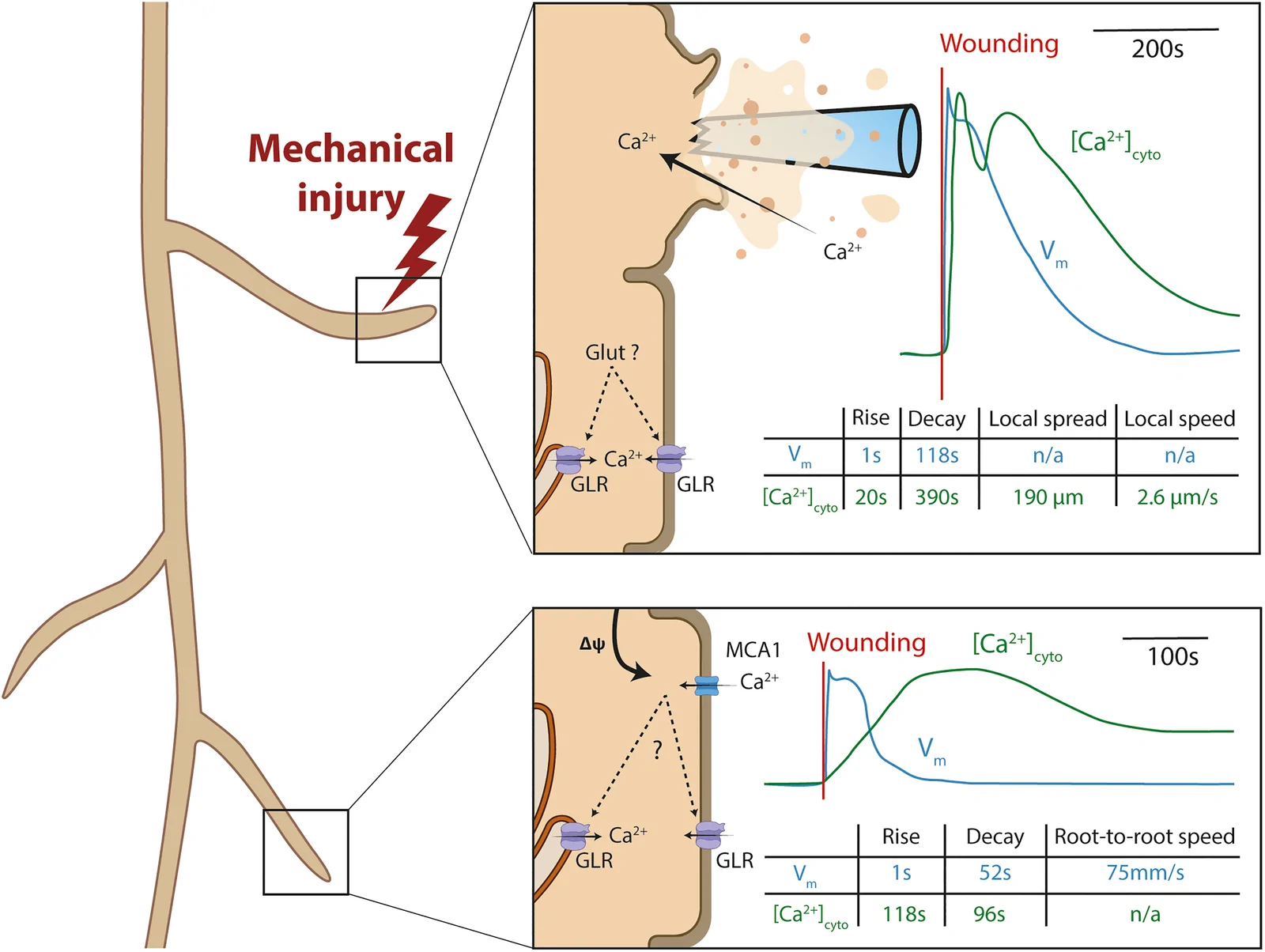
Proposed model of wound-induced signal propagation between lateral roots. Wounding of a lateral root generates a massive entry of ions into the cells, generating a high-amplitude Ca^2+^ transient and a concomitant depolarization in the wounded root. The local Ca^2+^ wave travels at low speed via cell-to-cell propagation and is sustained by a GLR3.3/3.6-dependent amplification system. The pressure drop generated by the wounding travels at high speed and reaches neighboring roots to activate MCA1 mechanosensitive channels, generating small-amplitude Ca^2+^ transients that are amplified by GLR3.3/3.6.

In the present study, we studied whether and how roots of *Arabidopsis* seedlings communicate with long-distance electrical and Ca^2+^ signals after wounding. Wounding of a lateral root evokes a Ca^2+^ transient and a depolarization, both locally and in adjacent lateral roots (Figs. 1 and 2). By examining the molecular components of the wound-mediated depolarization, we identified glutamate (Fig. 3), GLRs, and MCA1 mechanosensitive channels as crucial actors in this process (Figs. 4 to 7). Using optogenetic tools, we demonstrate that Ca^2+^ priming impairs glutamate but not pressure sensing (Fig. 8). Together, our findings indicate that root wounding provokes a drop in turgor pressure that rapidly propagates to neighboring lateral roots, activating mechanosensitive MCA1 cation channels and initiating a depolarization, subsequently amplified by GLRs (Fig. 9).

This hypothesis implies that the wound-induced depolarization response should scale with the magnitude of the pressure drop. Supporting this, the comparison between single-cell and large-scale wounding reveals that single-cell injury elicits a smaller, shorter-lived, and less far-reaching depolarization (Fig. 1). These findings are in agreement with Marhavý and colleagues (*12*), who laser ablated single cortex root cells while measuring extracellular root potentials with surface electrodes. Following single-cell wounding, surface potential changes were detectable within ∼200 μm of the wound site (*12*). In our study, intracellular membrane potential recording allowed us to detect small wound depolarization up to 2 mm, while large wounding-evoked depolarization traveled up to 5 mm (Fig. 1). Although this is sufficient to reach neighboring lateral roots, this distance is considerably shorter compared to what has been observed in the shoot, where wounding-associated depolarizations can travel several centimeters (*4*, *10*).

Because roots often grow in compact soils that slow insect movement, the local restriction of root wound signaling makes sense; insects that inflict root damage cannot travel through soil as easily as they move above ground. Thus, conveying information about an herbivory-induced attack to distant organs is likely less critical for roots than for shoots. For the same reason, one might also argue that electrical propagation does not need to be as fast as in the shoot. However, we measured a propagation speed that was very similar to leaf-to-leaf signaling speed (tens of millimeters per second) (*21*).

The cavitation bubble manometry technique indicates a pronounced decrease in turgor pressure in roots adjacent to the wounded root. Although this pressure drop is initiated immediately upon wounding, it reaches its maximum well after the depolarization of the epidermal root cells (Fig. 5). We therefore propose that mechanosensitive channels become saturated during the initial pressure decrease and, consequently, their activity does not scale with the full amplitude of the pressure change.

Another notable difference between wound-evoked signals in root and shoot tissue is the ability of seedlings’ roots to generate a distant wound-evoked depolarization even in the absence of GLR3.3 and GLR3.6. Given that the absence of these channels completely prevents wound-triggered electrical signals from spreading between leaves (*4*, *10*) and between the root and the shoot (*22*), one might expect a similar lack of long-distance signaling in roots. However, we detected wound-induced depolarizations—albeit with lower amplitude—in lateral roots adjacent to the injured site in mutants lacking functional GLRs. This divergence between root and shoot responses indicates a major difference in the channels/mechanisms implicated in long-distance wounding. Following root osmotic stress, glutamate levels have been observed to increase in the shoot, suggesting that glutamate waves can travel long distances (*23*). In our case, the depolarization propagating between lateral roots occurs too rapidly to be explained by passive diffusion. We thus propose that the pressure wave may instead trigger de novo glutamate signaling in the distal root.

Mechanosensitive channels have recently emerged as a key component in long-distance wound signaling in leaf-to-leaf communication. While the MSL10 anion channel appears to play a key role in wound-induced depolarization in the shoot (*8*), our findings indicate that the MCA1 Ca^2+^-permeable channel is the primary mechanosensor in the root. Nevertheless, mutating this channel could not completely wipe out distant wound-evoked depolarization, indicating that other channels contribute to this phenomenon. The participation of mechanosensitive channels and the observed speed of depolarization suggest that both root and shoot tissues likely rely on a shared wound signaling mechanism triggered by a change in turgor pressure, ultimately changing the membrane tension and activating mechanosensitive channels. However, different molecular players appear to decode this mechanical cue in each tissue.

More broadly, our data align with the squeeze cell hypothesis (*24*). According to this hypothesis, wounding induces an axial pressure change that propagates throughout the water column within vascular tissues (probably the xylem). This pressure variation then spreads radially to activate mechanosensitive channels and generate a GLR-dependent slow-wave potential (*25*).

## MATERIALS AND METHODS

### Growth conditions and plant material

*Arabidopsis thaliana* seeds were sterilized with 70% ethanol for 5 min and 6% sodium hypochlorite (NaClO) + Tween 20 for 10 min. After six washing steps with H_2_O, seeds were sown on agar plates [1.5% agar, 1.5% sucrose, and ½ MS (pH 5.8)] and stored at 4°C for at least 2 days. Afterward, plates were placed in a vertical position in a growth chamber (21°C; 120 μmol of photons/m^2^ per second) for 10 to 14 days. Plants expressing channelrhodopsins were grown under red light (*17*). The day before the experiment, samples were prepared as following: Small petri dish lids (⌀ = 3 cm) were coated with medical adhesive (Medical Adhesive B Liquid, VM 355-1, Ulrich AG), and then the seedlings were carefully removed from the growth medium with tweezers and placed in the glue, first sticking the tip of the root and then unrolling the root up to the hypocotyl (see fig. S1). The lids were then gently tapped against the workbench to ensure that all lateral roots were in contact with the glue and the samples were covered with recording solution containing 1 mM CaCl_2_, 1 mM KCl, and 5 mM MES/BTP (pH buffer balanced at pH 6). The next day, the plants were used for electrophysiology or imaging experiments.

The *osca1* mutant seeds were provided by Wang and colleagues (*26*), *35*S**::*GCaMP3* seeds by Gilroy and colleagues (*6*), *mca1* mutant seeds by Iida and colleagues (*27*), the *glr3.3/3.6* mutant seeds by Farmer and colleagues (*10*), and the lines expressing *AOS::NLS-3xVenus*, *ACS6::NLS-3xVenus*, and GCaMP3 in the *mca1/2* and in the *glr3.3/3.6* mutant background by Marhavý and colleagues (*9*). The constructs for expression of *Mb*Dio, ChR2 XXM2.0, and *Gt*ACR1 were described in (*19*).

### Electrophysiology

Petri dishes with immobilized seedlings (for preparation procedure, see the “Growth conditions and plant material” section and fig. S1) were placed on a microscope table (Akioskop 2FS, Zeiss, Germany) and illuminated with white light. To avoid photostimulation during optogenetic experiments, seedlings were illuminated with red light through a red light glass filter with a cutoff wavelength of 635 nm. Seedling roots were observed through a water immersion objective (W Plan-Apochromat, 40×/1.0, Zeiss). A capillary filled with 300 mM KCl and sealed with 2% agarose in 300 mM KCl was placed in the bath solution, serving as a reference electrode. This reference electrode was connected to the ground via an Ag/AgCl half-cell.

Epidermal cells of the lateral root EZ were impaled with microelectrodes that were made from borosilicate glass capillaries (inner/outer diameter = 0.56/1.0 mm; Hilgenberg, Germany), which were pulled using a horizontal laser puller (P2000, Sutter Instruments, CA, USA). The electrodes were filled with 300 mM KCl, mounted on the holder of a piezo-driven micromanipulator (MM3A, Kleindiek, Reutlingen, Germany), and connected to a headstage via an Ag/AgCl half-cell. The headstage was linked to a custom-made microelectrode amplifier (Ulliclamp 1). Electrical signals were low-pass filtered at 0.5 kHz with a dual low-pass Bessel filter (LPF 202A, Warner Instruments Corp., USA) and sampled at 1 kHz, while the WinWCP software controlled protocols (*28*). One or several pictures were taken following the recording to reconstruct the root network and calculate the distance between the recording electrode and the wounding capillary.

### Confocal microscopy and image processing

Experiments were conducted with ∼15-day-old *Arabidopsis* seedlings that express either *AOS::NLS-3xVenus* or *ACS6::NLS-3xVenus*. Lateral roots were crushed with fine forceps, and the plates were sealed again to avoid seedling dehydration. Five hours after wounding, seedlings were immobilized on a sample holder, and Venus expression was determined by acquiring images within the next 10 min. The fluorescence signals were detected with a confocal laser scanning microscope (Leica SP5, Leica Microsystems CMS) controlled by Leica LAS AF (version 2.7.3.9723, Leica Microsystems) with a dipping 40× objective. Venus was excited at 514 nm, and fluorescence was captured between 520 and 580 nm. Pictures were captured every 5 μm, forming a stack of 20 to 30 images. Fiji ImageJ-win64 software 60 was used for image processing. Stacks were projected on the *z* axis based on the maximum fluorescence, and nuclei were counted in a hand-drawn 300-μm circular region. The number of Venus-positive nuclei was averaged across roots within the same plant (typically two to three lateral roots per plant), and this value was used as a statistical unit.

### Wide-field fluorescence imaging

*Arabidopsis* seedlings expressing GCaMP3 under the 35*S* promoter (*6*) were excited with a light-emitting diode (LED) illumination system (pE-4000, CoolLED, Andover, UK) at 470 nm, and the light was passed through a bandpass filter (Semrock BrightLine; 472 ± 30 nm) on a dichroic mirror with a cutoff wavelength of 490 nm. The emission signal was detected using a band filter (Semrock BrightLine; 520 ± 35 nm) and a scientific complementary metal-oxide semiconductor camera (Teledyne Prime-BSI QuantEM, Photometrics). The illumination and image acquisition were controlled by the VisiView software (Visitron). Recordings were performed at a 0.2-Hz sampling frequency.

### Photostimulation of light-gated channels

For optogenetic experiments, plants were stimulated with light pulses of 470 nm for 1 s every 30 s with an intensity of 150 μmol/m^2^ per second. The constructs that were used for the expression of *Mb*Dio, ChR2 XXM2.0, and *Gt*ACR1 are described in (*19*).

### Wounding and local application of chemical stimuli

Glass capillaries were pulled using a horizontal puller (P2000, Sutter Instruments, CA, USA) and mounted on a micromanipulator. Pipettes with a tip diameter of ∼50 nm were used for single-cell wounding. For large wounds, pipette tips were broken to have a tip diameter of ∼10 μm, and capillaries were moved diagonally through at least 50% of the root cross section. For local applications, pipette tips were broken to have a tip diameter of ∼10 μm, and a pressure of 2 bars was applied with the PDES 02DX (NPI Electronics) to apply sorbitol during a period of 1 and 10 s for other chemicals. A 10-s application period corresponds to the ejection of 400 nl of the pipette solution.

### Intracellular pressure measurement

Individual plants were carefully removed from the agar and placed on a glass microscope slide with the root system immersed in a 100 mM MES-KOH solution. The distal ends of the plant (root tip and leaf tissue) were fixed to the microscope slide to prevent movement using a gel adhesive (tensive conductive adhesive gel, Natus Medical Inc., Wayzata, MN, USA) and allowed to equilibrate for 5 min. Using the methods of Brodersen *et al.* (*13*), cavitation microbubbles were generated in the epidermal cells of lateral roots within proximity of a second lateral root (<5 mm apart). Changes in cell turgor pressure were estimated by measuring the microbubble radius because the absolute pressure of the surrounding fluid constrains the growth of the microbubble. Microbubbles were generated with a modified laser ablation system (MicroPoint, Andor, Concord, MA, USA) mounted to the fluorescence port of a customized epifluorescence microscope and using a long-distance working objective lens (20× Mitutoyo America Corporation, Aurora, IL). Microbubble growth and dissolution dynamics were recorded at 1000 frames/s using a high-speed c-mount camera (model #acA640-750um, Basler Inc., Exton, PA). The light pulse was focused to a spot size of ∼5 μm and delivered 2.08 μJ of energy. Microbubbles were generated in lateral root epidermal cells for ∼10 min to measure the basal turgor pressure. Then, a glass microcapillary tube pulled to a tip of ∼5 μm was used to impale the epidermal cells of a nearby lateral root using a micromanipulator. We then generated microbubbles in the original lateral root for an additional 10 to 15 min to monitor changes in cell turgor in response to the wounding event. The resulting image sequences were then analyzed using ImageJ software to measure the diameter of the microbubbles.

### Analysis

All data were analyzed with homemade Python scripts available in the following repository: https://doi.org/10.5281/zenodo.20036842. Electrophysiology data were smoothed using a Gaussian filter (σ = 10) and downsampled to 10 Hz. The 10 s before the stimulation were used as the baseline, and their mean value was subtracted from the trace to calculate the depolarization amplitude. The rise and decay phases were fitted with a single exponential function, and constants were measured as the time necessary to reach 63.2 or 36.8% of the depolarization, respectively. A single exponential decay was used in Fig. 1 to correlate the depolarization amplitude with the distance between the wounding point and the recording site. This fitted model was then used to predict depolarization amplitude in long-distance wounding experiments, and the measured depolarization was normalized to the predicted value.

Imaging data were preprocessed using ImageJ to measure the background signal in a root-free area and the fluorescence variation at different hand-drawn region of interest along the root. The background fluorescence values were subtracted from all other values. The 60 s before the stimulation were used to determine the prestimulus value mean (*F*_0_), which was subtracted from all data points and used to normalize the changes in fluorescence signal (Δ*F*/*F*_0_). Data were then smoothed using a Savitsky-Golay filter, and the maximum Ca^2+^ signal was detected during the 60 s following the stimulation.

### Statistical analysis

Measurements were then exported to GraphPad Prism 8.0 for statistical analysis. Normality was tested before each test, and a parametric test or its nonparametric equivalent was performed according to data distribution.
