# Supplementary material for: MCA1 mechanosensitive channels enable fast communication of wound signals between lateral roots
Source: Sci Adv. 2026 Sep 25;12(39):eaef2202. doi: 10.1126/sciadv.aef2202 (PMC13614382; doi:10.1126/sciadv.aef2202)
Supplement: Supplementary file 1 [file sciadv.aef2202_sm.pdf]

Science Advances
AAAS

Supplementary Materials for

MCA1 mechanosensitive channels enable fast communication of wound signals between lateral roots

Angel Baudon *et al.*

Corresponding author: Angel Baudon, angel.baudon@uni-wuerzburg.de;
Rainer Hedrich, rainer.hedrich@suat-sz.edu.cn

Sci. Adv. **12**, eaef2202 (2026)
DOI: 10.1126/sciadv.aef2202

This PDF file includes:

Figs. S1 to S5
Tables S1 to S7

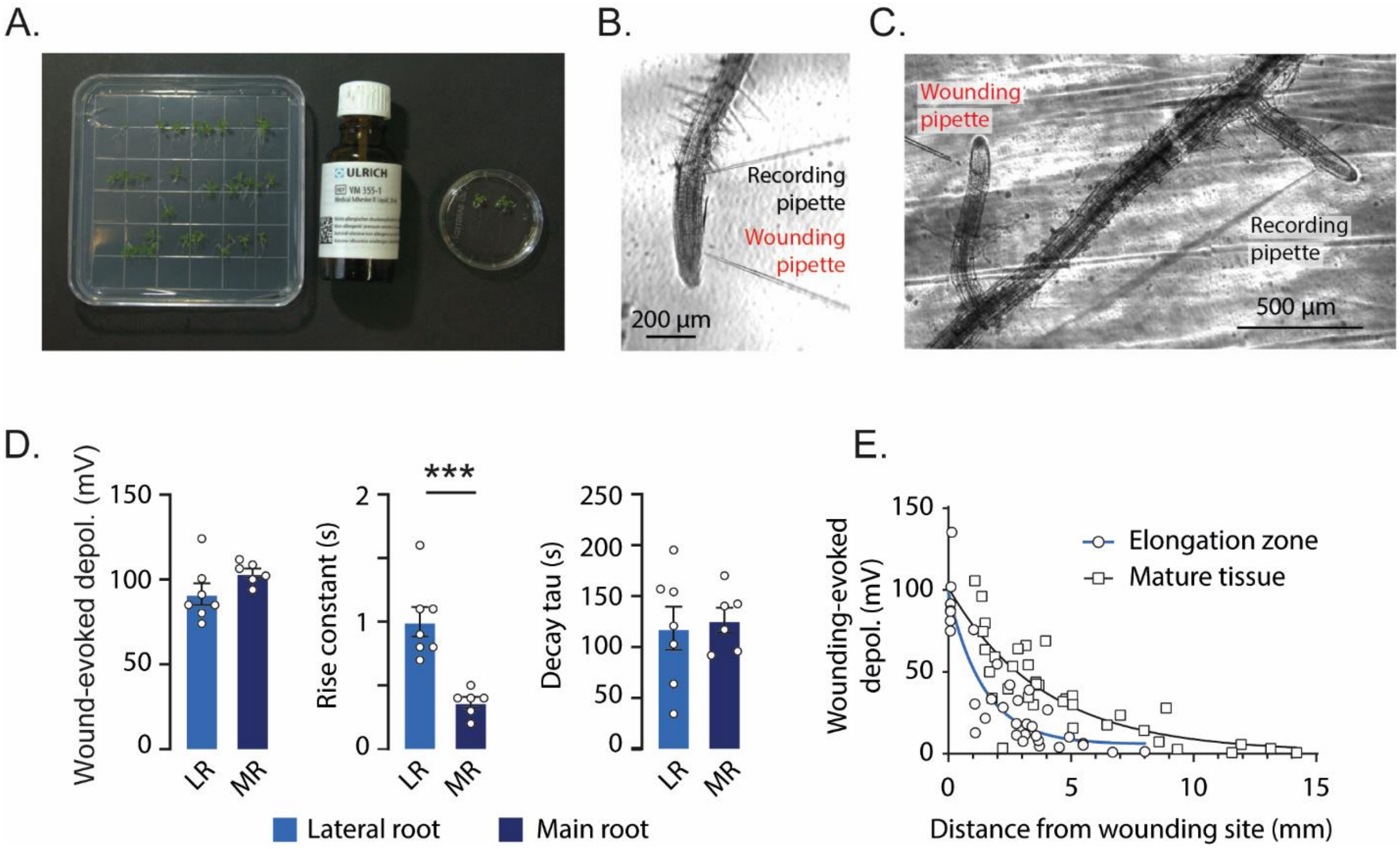

Fig. S1.

A. Agar plate with seedlings, glue, and a small Petri dish used as a chamber for membrane potential recordings. **B.** Lateral root of an Arabidopsis thaliana seedling with one microelectrode (upper capillary) used to impale an epidermal root cell, and a second glass pipette (lower capillary) used to wound the root tip. **C.** Two neighbouring lateral roots with the wounding pipette (left capillary tip) close to a lateral root, and the recording electrode (right capillary tip) impaling an epidermal cell of a neighbouring lateral root. **D.** Amplitude, rise, and decay constants of the depolarization triggered by a large wounding of a lateral root (LR), or of the main root (MR). **E**. Amplitude of the wounding-evoked depolarization plotted as a function of the distance between the wounded root and the recording point located in mature root tissues (squares), or in the elongation zone of another lateral root (circles). Each point represents an individual wound experiment. Data are expressed as mean across plants ± SEM. Detailed statistics can be found in the *Statistics Table*. ***$p<0.001$

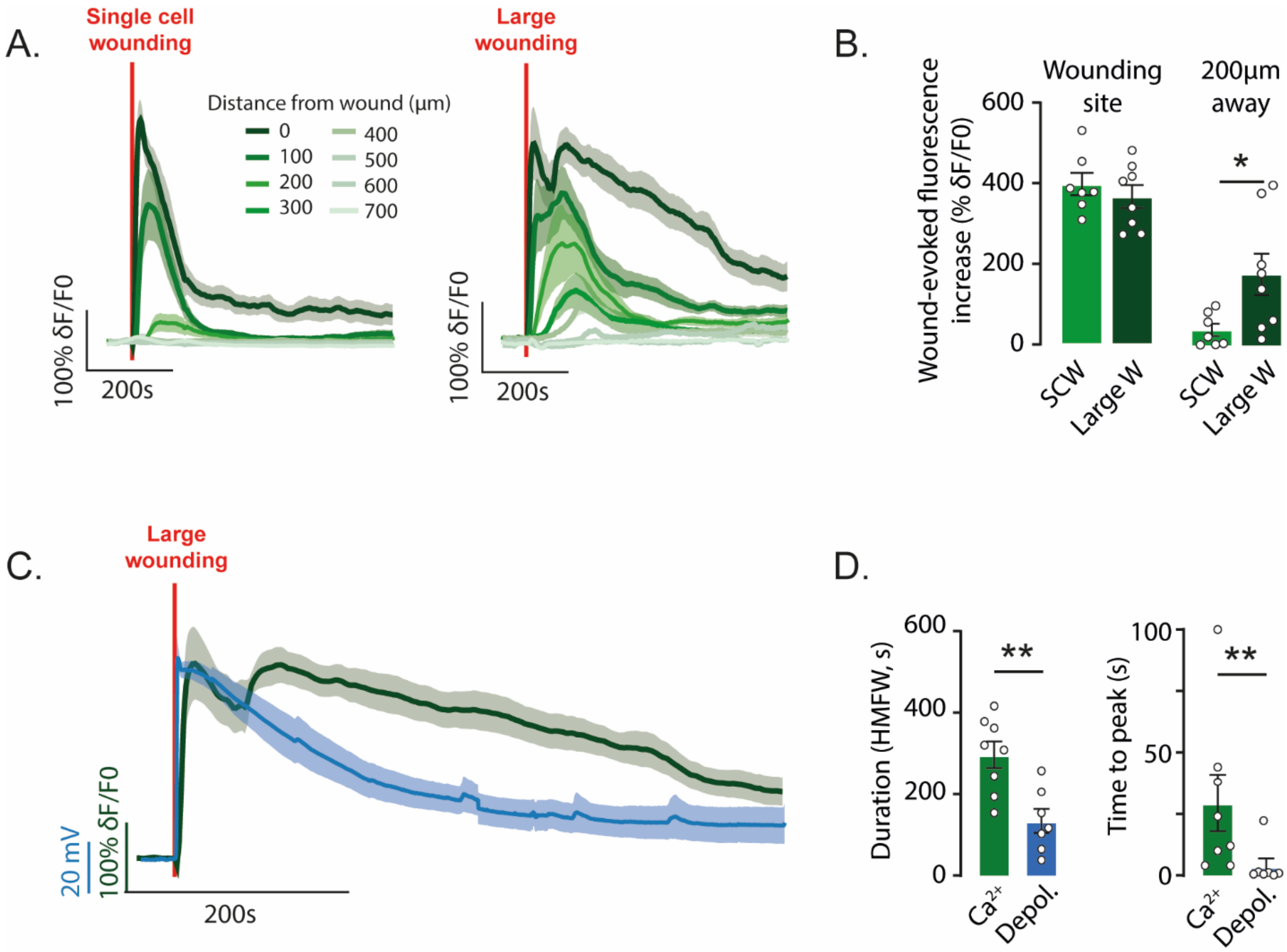

Fig. S2.

A. Average fluorescence levels of GCaMP3 measured at several distances from the site of a single-cell wounding (left, n=7), or a large wounding (right, n=8). Each trace represents an individual wound experiment. The red line indicates the time point of wounding. **B.** Quantification of GCaMP3 fluorescence at the wound site and 300μm away following single-cell (light green) or large wounding (dark green). **C.** Overlay of epidermal cell membrane potential (blue), and cytosolic Ca^{2+} level (green) following local wounding. Each trace represents an individual wound experiment. The red line indicates the time point of wounding. **D.** Duration measured as the half-maximum full width (HMFW, in seconds), and time to peak of the epidermal cell cytosolic Ca^{2+} level (green, "Ca^{2+}"), and membrane potential (blue, "Depol.") shown in C. Data are expressed as mean across plants ± SEM. Detailed statistics can be found in the *Statistics Table*. **p<0.01.

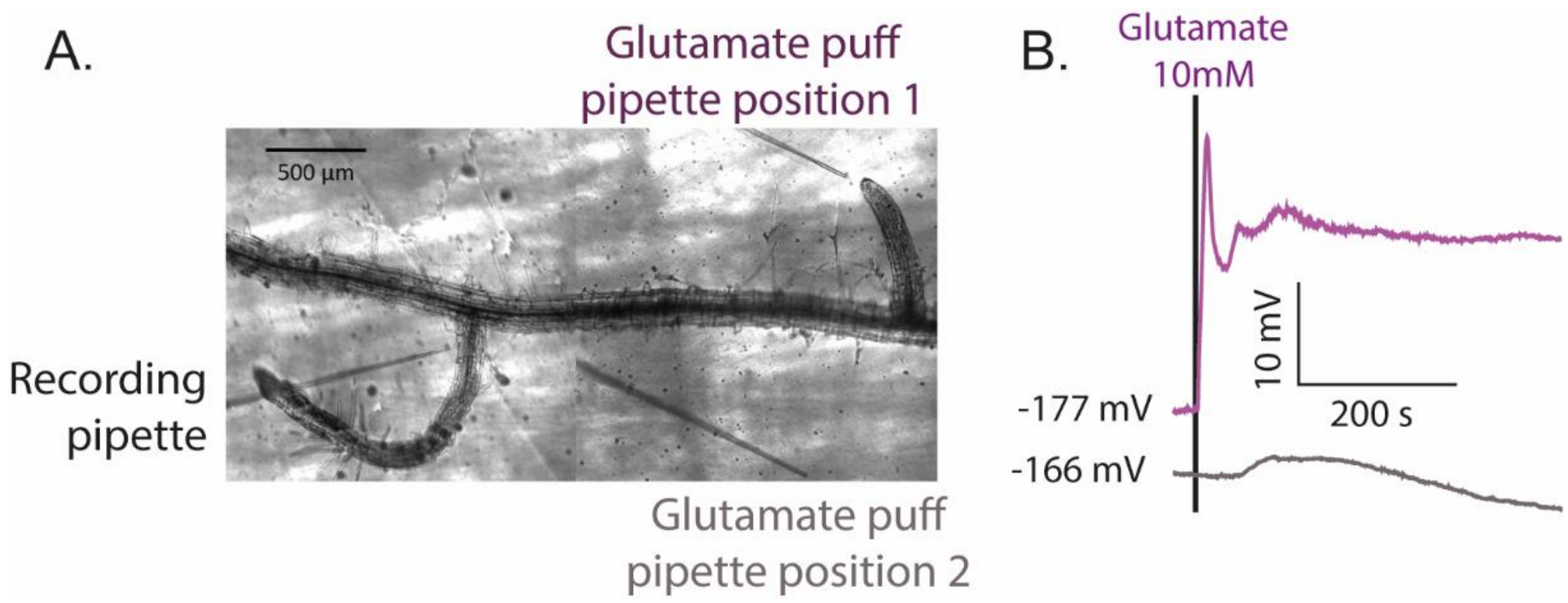

Fig. S3.

A-B. Control of local glutamate diffusion in the bath. **A.** Micrograph showing the position of the recording pipette, the puff application pipette on a distant root (position 1), and on a place without any root (position 2). **B.** Membrane potential recorded during local glutamate application at position 1 (purple upper trace) or at position 2 (grey lower trace), shown in panel A. The black line indicates the glutamate application time point. Scale = 500 µm.

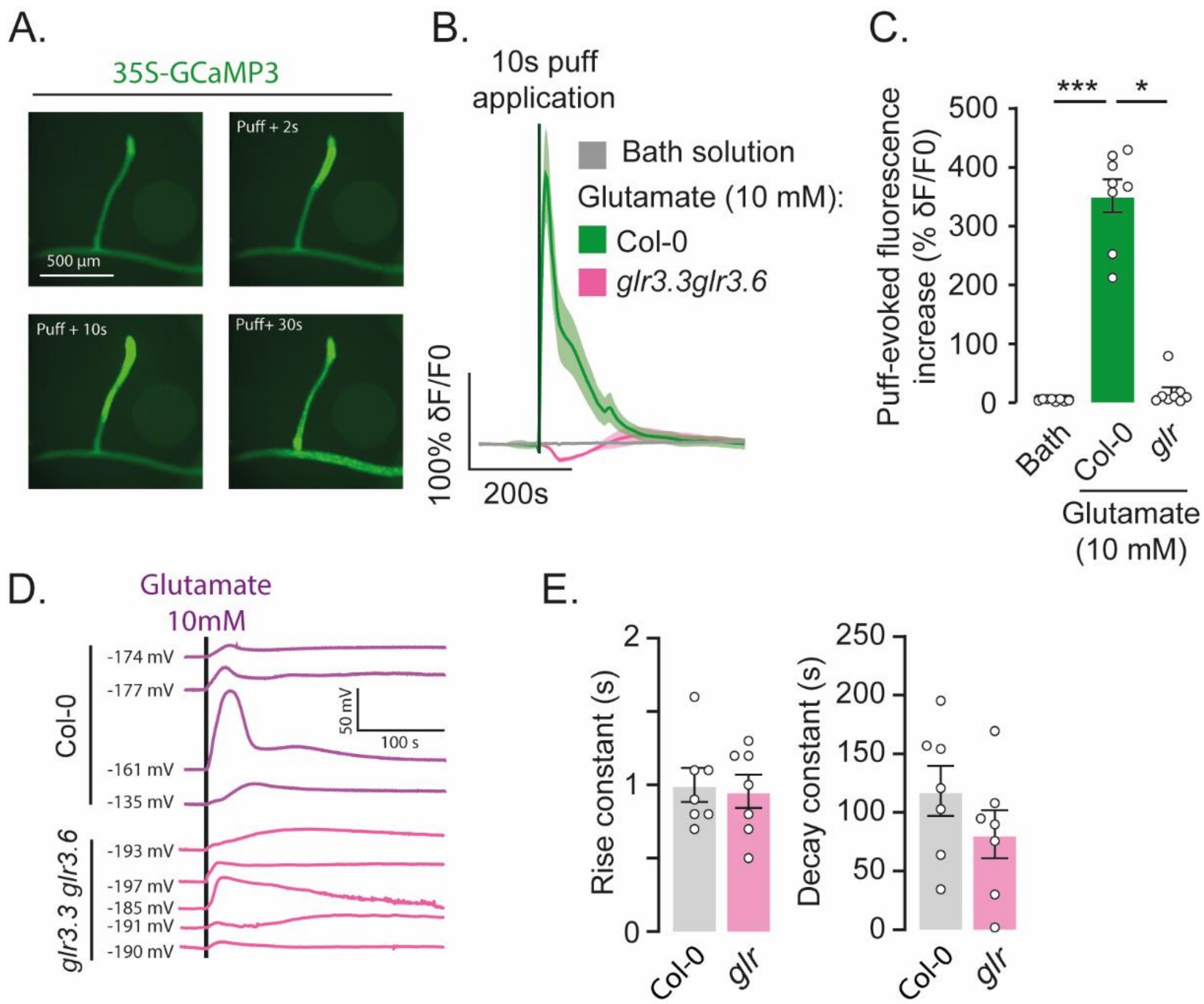

Fig. S4.

A. Fluorescence micrographs of a lateral root expressing GCaMP3 at different time points before, during, and after a puff application of glutamate (10mM). Scale = 500µm. **B.** Mean GCaMP3 fluorescence variation following puff application of the bath solution (grey), or of glutamate on control plants (Col-0, green), or on *glr3.3glr3.6* mutants (pink). The black line indicates the glutamate application time point. **C.** Quantification of GCaMP3 fluorescence following puff application of the bath solution (grey), or of glutamate on control plants (Col-0, green), or on *glr3.3glr3.6* mutants (pink). **D.** Membrane potential depolarization triggered by distant glutamate application in wild-type and *glr3.3/3.6* mutant plants. Each trace is from an individual wound experiment. The black line indicates the glutamate application time point. **E.** Rise and decay constant of wound-evoked depolarization recorded in *glr3.3/3.6* mutant and wild-type plants. Data are expressed as mean across plants ± SEM. Detailed statistics can be found in the *Statistics Table*. ***p<0.001

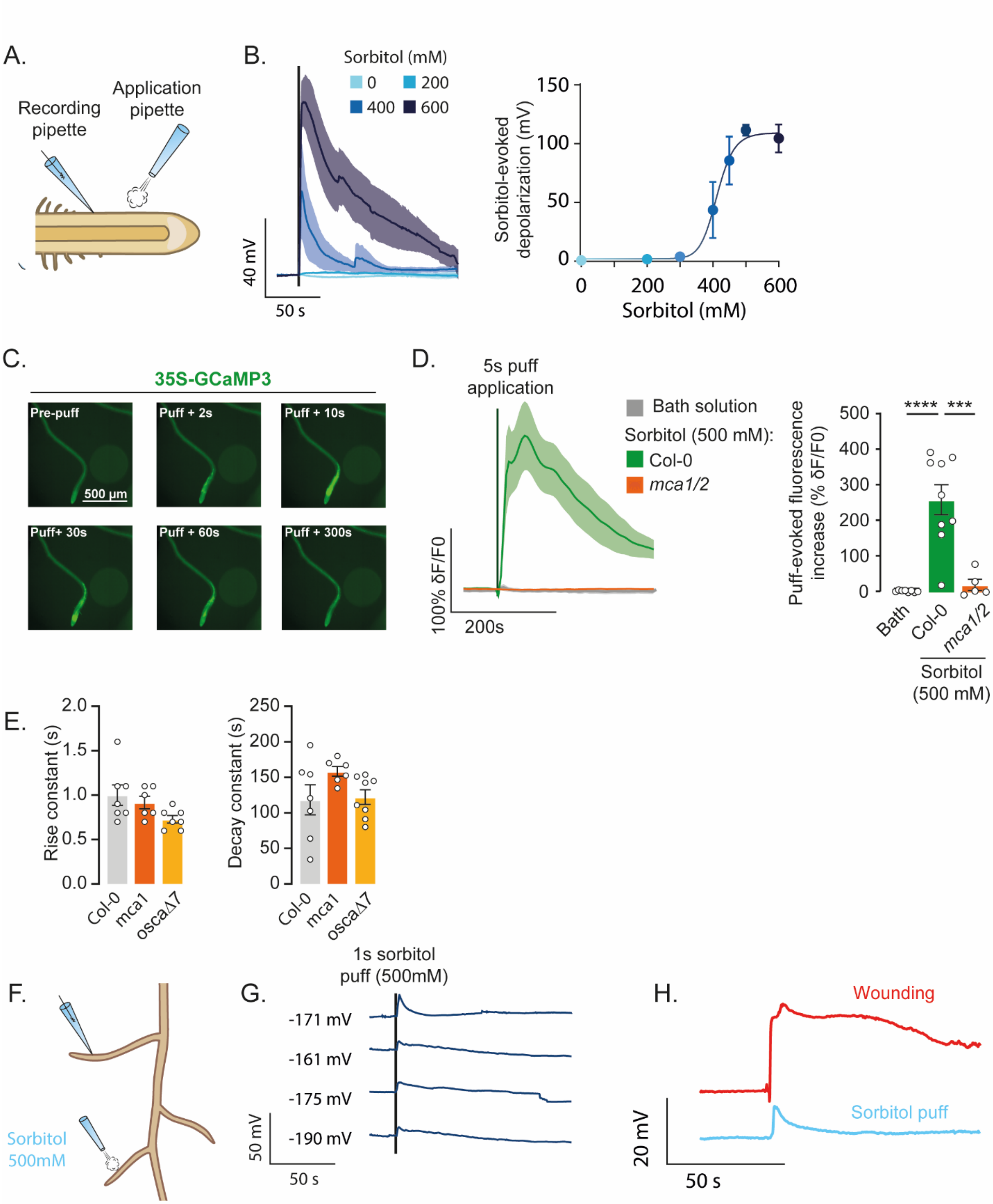

Fig. S5.

A. Epidermal root cells were impaled, and a solution containing different concentrations of sorbitol was backpressure-applied locally with a second glass pipette for 1s. **B.** Mean traces of sorbitol-evoked depolarization (left), and corresponding quantification (right). The sorbitol

application time point is indicated with the black line. Each trace comes from independent experiments. **C.** Fluorescence micrographs of a lateral root expressing GCaMP3 at different time points before, during, and after a puff application of sorbitol (500mM). Scale = 500µm. **D.** Left: Mean GCaMP3 fluorescence variation following puff application of the bath solution (grey), or of sorbitol on control plants (Col-0, grey), or on *mca1/2* mutants (orange). The black line indicates the sorbitol application time point. Right: corresponding quantification. **E.** Rise and decay constant of wound-evoked depolarization recorded in plants lacking functional mechanosensitive channels *mca1*, *osca1*, and in their controls (Col-0). Each data point comes from a different plant. **F.** Cartoon illustrating the local application of sorbitol on a lateral root and membrane potential recording of a neighbouring lateral root. **G.** Example traces of membrane potential depolarization evoked by sorbitol application on another distant lateral root. Each trace comes from a different plant; the number before the trace indicates the membrane potential at the beginning of the experiment, and the red line indicates the wounding time point. **H.** Comparison of the depolarizations evoked by applying sorbitol or wounding a distant root. The two recordings were performed in the same pair of roots. Data are expressed as mean across plants ± SEM. Detailed statistics can be found in the *Statistics Table*. ****$p<0.0001$

Table S1.

Numerical values and statistical analysis of data in Figure 1 and S1.

Fig	Statistical unit	Groups tested	Paired ?	n	Unit	Mean	SEM	Test	Statistic	p-value
**1C**	ACS6::NLS-3xVenus positive nuclei	Unwounded	No	11	/	11.230	1.261	Unpaired T-test	3.070	0.007
		Wounded		7		16.220	0.371			
**1C**	AOS::NLS-3xVenus positive nuclei	Unwounded	No	22	/	5.679	0.728	Mann Whitney test	24.500	<0.0001
		Wounded		21		14.190	1.235			
**1F**	Amplitude of wound-evoked depolarization	SCW	No	12	mV	49.440	4.715	Unpaired T-test	5.368	<0.0001
		Large wound		7		91.400	6.286			
**1F**	Rise constant of wound-evoked depolarization	SCW	No	12	s	0.775	0.107	Unpaired T-test	1.358	0.192
		Large wound		7		1.000	0.116			
**1F**	Decay constant of wound-evoked depolarization	SCW	No	12	s	21.450	6.764	Mann Whitney test	4.000	0.001
		Large wound		7		118.400	21.240			
**S1D**	Amplitude of wound-evoked depolarization	LR	No	6	mV	91.400	6.286	Unpaired T-test	1.715	0.114
		MR		7		103.800	2.650			
**S1D**	Rise constant of wound-evoked depolarization	LR	No	6	s	1.000	0.116	Unpaired T-test	4.821	0.001
		MR		7		0.367	0.042			
**S1D**	Decay constant of wound-evoked depolarization	LR	No	6	s	118.400	21.240	Unpaired T-test	0.309	0.763
		MR		7		126.300	30.200			

Fig	Statistical unit	Groups tested	n	Unit	Y0	Plateau	K	Half life	Tau	Span	R^2
**1J & S1E**	Exponential fit of wound-evoked depolarization as a function of distance	Elongation Z LW	34	mV/mm	98.220	5.745	0.001	1013.000	1461.000	92.480	0.811
		Elongation Z SCW	36		55.430	3.595	0.001	549.500	792.800	51.830	0.791
		Mature tissue	35		101.300	101.300	0.000	2697.000	3891.000	100.100	0.665

Table S2.

Numerical values and statistical analysis of data in Figure 2 and S2.

Fig	Statistical unit		Groups tested	Paired ?	n	Unit	Mean	SEM	Test	Statistic	p-value
**S2B**	Amplitude of wound-evoked Ca2+ transient	Wounding site	SCW	No	7	ΔF/F0	3.921	0.275	Unpaired T-test	0.775	0.452
			Large wound		8		3.613	0.283			
		200 µm away	SCW	No	7	ΔF/F0	0.072	0.013	Mann Whitney test	1.000	0.001
			Large wound		8		0.823	0.247			
**S2D**	Duration of wounding signal		Ca2+ transient	No	7	s	297.000	32.590	Unpaired T-test	3.670	0.003
			Depolarization		8		134.400	29.220			
**S2D**	Time to peak		Ca2+ transient	No	7	s	29.500	11.390	Mann Whitney test	4.000	0.003
			Depolarization		8		3.871	3.075			

Table S3.

Numerical values and statistical analysis of data in Figure 3 and S3.

Fig	Statistical unit	Group	Paired ?	n	Unit	Mean	SEM	Bottom	Top	LogEC50	HillSlope	EC50	Span	R^2
3B	Glutamate-evoked depolarization	0 mM	No	4	mV	3.513	0.826	5.960	63.310	4.714	0.436	51777.000	57.350	0.619
		1 mM		4		10.450	0.934							
		5 mM		7		38.550	8.769							
		10 mM		12		65.030	7.094							
		100 mM		5		58.620	5.437							
3C	ATP-evoked depolarization	0.001 mM	No	4	mV	4.546	0.241	2.264	0.521	128.000	1.639	0.215	125.800	0.944
		0.01 mM		5		9.645	4.259							
		0.1 mM		5		28.830	5.124							
		1 mM		6		54.560	3.483							
		10 mM		3		96.630	3.180							
		100 mM		5		113.900	7.888							
3D	H2O2-evoked depolarization	0 mM	No	2	mV	0.628	0.299	8.096	~ 35.37	63.910	~ 3.110	~ 0.4927	55.810	0.743
		0.01 mM		5		3.253	0.989							
		0.1 mM		6		14.620	3.659							
		1 mM		9		20.310	4.983							
		10 mM		3		64.650	11.740							
		100 mM		3		63.160	12.640							

Table S4.

Numerical values and statistical analysis of data in Figure 4 and S4.

Fig	Statistical unit	Groups tested	Paired ?	n	Unit	Mean	SEM	Test	Statistic	p-value	Post-hoc	p-value
4C	Amplitude of glutamate-evoked depolarization	Col-0	No	12	mV	65.030	7.094	Unpaired t test	4.400	3.00E-04		
		glr3.3/glr3.6		10		24.790	5.253					
4F	Amplitude of local wound-evoked depolarization	Col-0	No	7	mV	91.400	6.286	Mann Whitney test	28.000	>0.9999		
		glr3.3/glr3.6		8		78.730	12.870					
4I	Amplitude of distant wound-evoked	Ctrl	No	27	/	0.953	0.123	Mann Whitney test	56.000	5.80E-03		
		glr3.3glr3.6		10		0.410	0.093					
S4C	Amplitude of glutamate-evoked Ca2+ transient	Bath solution	No	8	ΔF/F0	0.042	0.008	Kruskal-Wallis ANOVA	16.810	2.00E-04	Dunnett´s MC vs Glut	1.00E-04
		Glutamate		8		3.515	0.278					
		Glut on glr		8		0.172	0.090					1.04E-02
S4E	Rise constant of wound-evoked depolarization	Col-0	No	7	s	1.000	0.116	Unpaired T-test	0.265	0.795		
		glr3.3/glr3.6		7		0.957	0.113					
S4E	Decay constant of wound-evoked depolarization	Col-0	No	7	s	118.400	21.240	Unpaired t test	1.250	0.235		
		glr3.3/glr3.6		7		81.440	20.520					

Table S5.

Numerical values and statistical analysis of data in Figures 5, 6, and S5.

Fig	Statistical unit	Group	Paired ?	n	Unit	Mean	SEM	Bottom	Top	LogEC50	HillSlope	EC50	Span	R^2
S5C	Sorbitol-evoked depolaization	0 mM	No	2	mV	0.628	0.299	2.122	15.900	109.400	412.500	2.615	107.200	0.693
		200 mM		5		1.688	0.657							
		300 mM		4		3.861	2.376							
		400 mM		5		43.600	23.890							
		450 mM		6		85.740	20.400							
		500 mM		4		111.500	4.625							
		600 mM		4		104.600	11.970							

Fig	Statistical unit	Groups	Paired ?	n	Unit	Mean	SEM	Test	Statistic	p-value	Post-hoc	p-value
5C	Wound-evoked depolarization propagation speed		No	6	mm/s	74.360	18.220	/	/	/	/	/
6C	Amplitude of sorbitol-evoked depolarization	Col-0	No	4	mV	111.500	4.625	Kruskal-Wallis test	8.226	0.016	Dunnett´s MC vs Col-0	
		mca1		10		23.120	8.759					0.012
		osca1.1-1.7		12		44.700	15.690					0.022
6F	Amplitude of local wound-evoked depolarization	Col-0	No	7	mV	92.080	6.005	Kruskal-Wallis test	2.450	0.307	/	/
		mca1		6		98.850	2.679					
		osca1.1-1.7		8		94.000	5.580					
6I	Amplitude of distant wound-evoked depolarization	Ctrl	No	27	/	0.953	0.123	Mixed-model ANOVA	Predicted vs Measured: 3.975<br>Genotype: 0.2975<br>Interaction: 5.198	Predicted vs Measured: 0.0635<br>Genotype: 0.5930<br>Interaction: 0.0367	Sidak's MC	
		mca1		11		0.518	0.058					0.029
		osca1		11		0.921	0.124					0.968
S5F	Amplitude of sorbitol-evoked Ca2+ transient	Bath solution	No	8	ΔF/F0	0.038	0.009	One way ANOVA	23.720	<0.0001	Dunnett´s MC vs Sorb	1.00E-04
		Sorbitol		9		2.603	0.422					
		Sorb on mca1		5		0.217	0.157					1.00E-04
S5G	Rise constant of wound-evoked depolarization	Col-0	No	7	s	1.000	0.116	One way ANOVA	2.926	0.081		
		mca1		6		0.917	0.070					
		osca1.1-1.7		8		0.729	0.042					
S5G	Decay constant of wound-evoked depolarization	Col-0	No	7	s	118.400	21.240	One way ANOVA	2.142	0.146		
		mca1		6		158.500	6.874					
		osca1.1-1.7		8		122.400	10.310					

Table S6.

Numerical values and statistical analysis of data in Figure 7.

Fig	Statistical unit	Groups tested	n	Unit	Y0	Plateau	K	Half life	Tau	Span	R^2
7B	Exponential fit of wound-evoked Ca2+ transient as a function of distance	Ctrl	8	ΔF/F0/mm	3.776	9.83E-07	0.004	192.800	278.100	3.776	0.613
		mca1/2	12		4.145	2.41E-08	0.003	214.300	309.200	4.145	0.538
		glr3.3glr3.6	9		3.824	4.02E-02	0.012	58.270	84.060	3.784	0.873

Fig	Statistical unit	Groups tested		Paired ?	n	Unit	Mean	SEM	Test	Statistic	p-value	Post-hoc	p-value
7C	Amplitude of wound-evoked Ca2+ transient	Wounding site	Ctrl	No	8	ΔF/F0	3.613	0.283	One-Way ANOVA	0.330	0.721	/	/
			mca1/2		12		4.140	0.579					
			glr3.3glr3.6		9		3.805	0.325					
		200 µm away	Ctrl	No	8	ΔF/F0	1.750	0.509	Kruskal Wallis test	14.790	0.001	Dunnett´s MC vs Ctrl	
			mca1/2		12		2.239	0.464					1.000
			glr3.3glr3.6		9		0.229	0.111					0.007
7E	Amplitude of local wound-evoked Ca2+ transient	Ctrl		No	10	ΔF/F0	0.034	0.006	One-Way ANOVA	10.100	0.001	Dunnett´s MC vs Ctrl	
		mca1/2			8		-0.004	0.008					0.001
		glr3.3glr3.6			8		0.008	0.005					0.012

Table S7.

Numerical values and statistical analysis of data in Figure 8.

Fig	Statistical unit	Groups tested	Paired ?	n	Unit	Mean	SEM	Test	Statistic	p-value	Post-Hoc	p-value
**8C**	Amplitude of glutamate-evoked depolarization	Retinal	No	9	mV	36.670	6.012	One-Way ANOVA	9.828	0.001	Dunnett´s MC vs Retinal	
		ChR-XXM		13		8.612	1.945					0.008
		ACR1		7		48.150	13.690					0.446
**8E**	Amplitude of sorbitol-evoked depolarization	Retinal	No	6	mV	54.870	12.300	One-Way ANOVA	0.364	0.700	/	/
		ChR-XXM		6		50.900	7.963					
		ACR1		7		43.330	9.088					
**8H**	Amplitude of wound-evoked depolarization	Retinal	No	7	mV	83.590	5.987	Kruskal Wallis test	3.642	0.164	/	/
		ChR-XXM		9		27.310	6.163					
		ACR1		5		94.110	6.462					
**8J**	Amplitude of wound-evoked depolarization (norm to control)	Retinal	No	7	/	1.133	0.177	Unpaired T-test	2.641	0.023		
		ChR-XXM		9		0.345	0.123					
